# Strigolactone Levels in Dicot Roots Are Determined by an Ancestral Symbiosis-Regulated Clade of the *PHYTOENE SYNTHASE* Gene Family

**DOI:** 10.3389/fpls.2018.00255

**Published:** 2018-03-01

**Authors:** Ron Stauder, Ralf Welsch, Maurizio Camagna, Wouter Kohlen, Gerd U. Balcke, Alain Tissier, Michael H. Walter

**Affiliations:** ^1^Department of Cell and Metabolic Biology, Leibniz-Institute of Plant Biochemistry, Halle, Germany; ^2^Institute of Biology II, Faculty of Biology, University of Freiburg, Freiburg, Germany; ^3^Laboratory of Molecular Biology, Department of Plant Sciences, Wageningen University, Wageningen, Netherlands

**Keywords:** carotenoids, apocarotenoids, strigolactones, symbiosis, arbuscular mycorrhiza, *Medicago truncatula*, *Solanum lycopersicum*

## Abstract

Strigolactones (SLs) are apocarotenoid phytohormones synthesized from carotenoid precursors. They are produced most abundantly in roots for exudation into the rhizosphere to cope with mineral nutrient starvation through support of root symbionts. Abscisic acid (ABA) is another apocarotenoid phytohormone synthesized in roots, which is involved in responses to abiotic stress. Typically low carotenoid levels in roots raise the issue of precursor supply for the biosynthesis of these two apocarotenoids in this organ. Increased ABA levels upon abiotic stress in Poaceae roots are known to be supported by a particular isoform of phytoene synthase (PSY), catalyzing the rate-limiting step in carotenogenesis. Here we report on novel *PSY3* isogenes from *Medicago truncatula* (*MtPSY3*) and *Solanum lycopersicum* (*SlPSY3*) strongly expressed exclusively upon root interaction with symbiotic arbuscular mycorrhizal (AM) fungi and moderately in response to phosphate starvation. They belong to a widespread clade of conserved PSYs restricted to dicots (dPSY3) distinct from the Poaceae-PSY3s involved in ABA formation. An ancient origin of dPSY3s and a potential co-evolution with the AM symbiosis is discussed in the context of PSY evolution. Knockdown of *MtPSY3* in hairy roots of *M. truncatula* strongly reduced SL and AM-induced C_13_ α-ionol/C_14_ mycorradicin apocarotenoids. Inhibition of the reaction subsequent to phytoene synthesis revealed strongly elevated levels of phytoene indicating induced flux through the carotenoid pathway in roots upon mycorrhization. d*PSY3* isogenes are coregulated with upstream isogenes and downstream carotenoid cleavage steps toward SLs (*D27*, *CCD7*, *CCD8*) suggesting a combined carotenoid/apocarotenoid pathway, which provides “just in time”-delivery of precursors for apocarotenoid formation.

## Introduction

**FIGURE 1:**
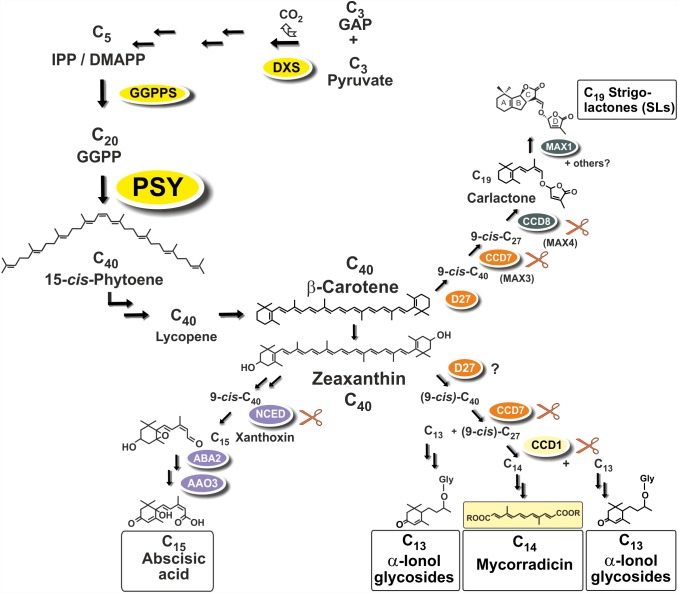
Apocarotenoid biosynthetic pathways in roots and their carotenoid precursor supply through phytoene synthase (PSY). The simplified scheme indicates three key steps of carotenoid biosynthesis marked in yellow [1-deoxy-D-xylulose 5-phosphate synthase, DXS (MEP-pathway, horizontal arrows to the left), GGPP synthase, GGPPS, PSY, vertical arrows] and other conversions leading to β-carotene and zeaxanthin apocarotenoid precursors. Cleavage by carotenoid cleavage dioxygenases (CCDs) or 9-*cis*-epoxy-carotenoid cleavage dioxygenases (NCEDs) starts from 9-*cis* isomerized substrates by a single or by sequential double cleavage reactions followed by modification steps leading to various apocarotenoid products (boxed). A potential involvement of D27 in C_13_/C_14_ apocarotenoid biosynthesis is still unresolved indicated by a question mark.

Strigolactones (SLs) and abscisic acid (ABA) are carotenoid-derived phytohormones (apocarotenoids) synthesized through enzymatic oxidative tailoring of carotenoid precursors (Walter and Strack, 2011; McQuinn et al., 2015; Nisar et al., 2015; Hou et al., 2016). They are most abundantly formed in roots but are also synthesized in other organs. SLs operate as branching inhibitor hormones in the regulation of shoot architecture (Bennett and Leyser, 2014; Al-Babili and Bouwmeester, 2015). SLs are also exuded from roots into the rhizosphere, where they govern symbiotic relationships with beneficial soil fungi and soil bacteria (Andreo-Jimenez et al., 2015; López-Ráez et al., 2017). The multifaceted roles of ABA encompass adaptation to salt, drought and other abiotic stress conditions (Nambara and Marion-Poll, 2005). Biogenesis of SLs involves two sequential cleavage reactions by carotenoid cleavage dioxygenases (CCD7, CCD8) acting on β-carotene after its enzymatic isomerization into the 9-*cis* configuration by an enzyme called D27. Further modifications of the carlactone intermediate involve one or more oxidative steps by P450 enzymes called MAX1 leading to canonical and non-canonicals SLs (**Figure 1**; Alder et al., 2012; Al-Babili and Bouwmeester, 2015). The ABA precursor xanthoxin results from a single cleavage reaction on an epoxidized 9-*cis*-zeaxanthin isomer (**Figure 1**; Nambara and Marion-Poll, 2005).

Arbuscular mycorrhiza (AM) supporting plant mineral nutrition is an ancestral and arguably the most prevalent mutualistic symbiosis on earth (Parniske, 2008). It develops between the roots of most terrestrial plants and fungal species of the phylum Glomeromycota. Fungal hyphae colonize plant roots and this process is promoted by a hyphal branching stimulation activity of SLs exuded from roots (Akiyama et al., 2005). Synthesis of SLs in roots is stimulated by phosphate starvation correlated with elevated colonization in nutrient starvation conditions (López-Ráez et al., 2008, 2011b; Balzergue et al., 2011, 2013; Foo et al., 2013; Carbonnel and Gutjahr, 2014). In root cortex cells, hyphae can form highly branched symbiotic organs called arbuscules (Harrison, 2012; Gutjahr and Parniske, 2013; Recorbet et al., 2013). Arbuscules constitute the symbiotic interface for nutrient exchange between the plant and the fungus.

Two additional types of root apocarotenoids occur, except for trace amounts in some species, only upon colonization by these AM fungi. They emerge in later stages of the symbiosis and are localized to arbusculated cells (Fester et al., 2002a). The first type comprises various glycosylated C_13_ α-ionols derivatives (formerly called cyclohexenone derivatives, **Figure 1**; Maier et al., 1995; Fester et al., 2002a; Schliemann et al., 2008; Walter and Strack, 2011). The second is a dicarboxylic acid polyene called mycorradicin (C_14_), which retains enough of the carotenoid chromophore to be a “yellow pigment”—a term and coloration known to many AM researchers as an indicator of mycorrhizal colonization (**Figure 1**; Klingner et al., 1995). Silencing *CCD1* expression in mycorrhizal hairy roots revealed a sequential two-step cleavage mechanism comparable to SL biogenesis, in which CCD1 acts as the second cleavage enzyme (**Figure 1**; Floss et al., 2008b). Subsequent studies in tomato have shown that CCD7 is involved in the first cleavage reaction in both SL and mycorrhizal C_13_/C_14_ apocarotenoid biosynthesis (Vogel et al., 2010).

Phytoene synthase (PSY) catalyzes the first committed and rate-limiting step in carotenoid biosynthesis condensing two molecules of the C_20_-prenyl phosphate geranylgeranyl diphosphate (GGPP) into C_40_-phytoene (**Figure 1**). The first member of the angiosperm *PSY* gene family (*PSY1*) was cloned from tomato fruits and its expression correlated with lycopene accumulation (Bartley et al., 1992; Ray et al., 1992). A second tomato gene (*PSY2*) with preferential expression in photosynthetic leaves was identified subsequently (Bartley and Scolnik, 1993). In two Poaceae species (rice and maize), a third type of *PSY* gene was described, named PSY3, whose expression was induced by drought and salt treatment, compatible with a specialized role in providing precursors for the formation of ABA (Li et al., 2008; Welsch et al., 2008). Three *PSY* genes were also identified from the dicot cassava (*Manihot esculenta*). Surprisingly, the one *PSY* gene out of the three cassava *PSY* homologs most closely related to the *PSY3* of the Poaceae (*MePSY3*) did not exhibit any transcriptional response to abiotic stress treatments (Arango et al., 2010). *MePSY3* transcripts were virtually absent in all cassava tissues and conditions tested (Arango et al., 2010). Since distinct isoforms of PSY seem to be associated with organ- or tissue-specific production of carotenoids, this raises the issue of the potential function of dicot *PSY3* genes and their connection to organ- or condition-specific accumulation of carotenoids or apocarotenoids.

Continuing earlier work on AM-mediated regulation of (apo)carotenoid precursor biosynthetic isogenes in the MEP pathway (*DXS2*; Walter et al., 2000, 2002, 2007; Floss et al., 2008a), we searched for *PSY* isogenes potentially regulated by AM fungi to provide carotenoid precursors for the biosynthesis of SL and C_13_/C_14_ apocarotenoids. Here we show that in two dicots from different plant families (*Medicago truncatula*, *Solanum lycopersicum*) *PSY* isogenes closely related to *MePSY3* are strongly regulated in roots almost exclusively during colonization by AM fungi and to a lesser extent by phosphate starvation. The PSY3 proteins encoded are members of a previously unrecognized, highly conserved dicot-specific class of PSY3 enzymes. This newly identified PSY3 clade is discussed in the context of diversity, expression profiles and evolution of the plant *PSY* gene family extending an earlier preliminary account on this topic (Walter et al., 2015).

## Materials and Methods

### Plant Material and Growth Conditions

*Medicago truncatula* GAERTN. var. Jemalong cv. A17 seeds were scarified with 1 ml sulfuric acid for 5–8 min, washed 5× in water, placed on moist filter paper and stored for 3 days at 4°C in the dark. Prior to transfer on expanded clay, seedlings were kept for 1 day each in the dark and in light at room temperature. Plants were grown on expanded clay substrate (Lecaton, Lamstedt, Germany, particle size 2–5 mm) in a greenhouse (16 h light, 8 h dark at 23°C/18°C and 50% relative humidity). Plants were irrigated twice a week with deionized water and fertilized once in the first 2 weeks and twice per week subsequently with 10 ml half-strength Long Ashton fertilizer.

*Solanum lycopersicum* seeds cv. M82 (courtesy of H. Klee, University of Florida, United States) were sterilized in 70% ethanol for 15 min followed by a 15 min treatment with 6% sodium hypochlorite and washed 3× in water. Seeds were germinated on expanded clay for 7 days in a greenhouse. Seedlings were transferred to pots and grown under similar irrigation and fertilizing conditions as in the *M. truncatula* experiments. Conditions for harvesting tomato leaves and fruits have been described (Paetzold et al., 2010).

### Root Treatments: Phosphate Starvation, Fungal Inoculation, Salt and Drought Treatments, and Norflurazon Application

For phosphate starvation treatments the inorganic phosphate (Pi) source in the Long Ashton fertilizer (NaH_2_PO_4_.H_2_O) was reduced in various steps from 100% (6.6 mM Pi, full Pi supply, control) down to 20, 10, 5, or 2% residual Pi supply as indicated. Spores of the AM fungus *Rhizophagus irregularis* were obtained from Symplanta (Munich, Germany). Mycorrhizal inoculum was produced on leek as a trap culture in expanded clay and mixed 20:80 (w/w) with expanded clay prior to use. At least three *M. truncatula* or *S. lycopersicum* biological replicates were cultivated under the various phosphate starvation, mycorrhizal, saline, drought, or combined treatments for about 6 weeks before harvest. For salt treatment 1 on plants raised on 20% phosphate an aqueous solution of 150 mM NaCl (40 ml) was applied 2 h prior to harvest. Salt treatment 2 involved two sequential treatments with the same NaCl solution at 5 and 2 h prior to harvest. Drought stress involved withholding irrigation for 6 days (drought 1) or 10 days (drought 2) before harvest. Norflurazon treatments were started 8 days before harvest and performed by applying 40 ml of an aqueous 25 mg l^-1^ norflurazon solution to the substrate every second day. Controls received the same volume of water. Each biological replicate consisted of a single plant. The outer parts of the roots (about one-third of the whole root system) were cut off and fractions from the central part of the root system were used for RNA isolation and (apo)carotenoid analysis.

### Real-Time qRT-PCR

RNA isolation from plant material was done with the RNeasy Mini Plant Kit (Qiagen, Hilden, Germany) according to the manufacturer’s instructions. To synthesize cDNA from 0.5 μg of RNA the RevertAid First Strand cDNA Synthesis Kit (Thermo Fisher Scientific) was used according to the manufacturers’ instructions. The mixture underwent a temperature regime of 60 min at 42°C, and 5 min at 70°C. The cDNA obtained was diluted 10× before serving as a template for RT-PCR. qRT-PCR was performed on a CFX96 system (Bio-Rad, Munich, Germany) using SYBR Green I. Three microliters of diluted cDNA was mixed with 2 μl of 5× EvaGreen qPCR mix II (Bio&Sell, Feucht, Germany), 0.1 pmol each of forward and reverse primer adding dH_2_O for a total volume of 10 μl. Primers are listed in Supplementary Table S2. Two technical replicates were done for each biological replicate. Target gene values were normalized to *MtEF1α* and *SlEF1α* constitutively expressed reference genes. Normalized expression was calculated by the 2^-ΔCT^ method (Schmittgen and Livak, 2008).

### Assay for SlPSY3 Enzyme Activity

Prerequisites of the enzyme assay and the respective controls were the expression and purification of recombinant Arabidopsis GGPP synthase 11 for *in situ* substrate generation, of ZmPSY1 as a positive control and of a codon-optimized synthetic SlPSY3. The cDNA of AtGGPPS11 (from base 238 to 1189 of accession AK227130, Ruiz-Sola et al., 2016) was inserted into vector pETDuet^TM^-1 (Novagen) to be expressed in *E. coli* BL21 cells (Novagen) as N-terminal 6×His fusion as described (Kloer et al., 2006). Cells were resuspended in 10 ml buffer A [20 mM Tris/HCl, pH 8.0, 100 mM NaCl, 10 mM MgCl_2_, 10 % (v/v) glycerol], disrupted with a French Press (Amicon). 6^∗^His-AtGGPS11 was purified with TALON Co^2+^ resin (Clontech) according to the manufacturer’s instruction. The cDNA of ZmPSY1 (accession U32636) and the codon-optimized SlPSY3 were truncated by the sequence encoding the transit peptide (ZmPSY1: 50 amino acids; SlPSY3: 20 amino acids) using PCR amplification with mutagenized primers and subcloned into the vector pCOLD1 (Takara-Clontech) to yield N-terminal 6×His fusions. Expression and purification of SlPSY3 was performed as described (Welsch et al., 2008).

Five micrograms of purified 6×His-PSY and 7.5 μg of purified 6^∗^His-AtGGPPS11 were incubated in 800 μl of reaction buffer [100 mM Tris/HCl, pH 7.6, 2 mM MnCl_2_, 1 mM Tris(2-carboxyethyl)phosphine hydrochloride, 0.08% (v/v) Tween 80, 10 mM MgCl_2_, and 20% (v/v) glycerol]. The reaction was initiated with 40 μM DMAPP and 60 μM IPP (Isoprenoids Lc) and incubated over night at room temperature. Assays were extracted with CHCl_3_:MeOH (2:1, v/v) and the chloroform phase was analyzed on an UFPL Shimadzu Prominence system coupled with a PDA [C30-RP YMC carotenoid, 150 mm × 3 mm, S-5 μm; solvent systems B: MeOH/*tert*-butyl-methyl ether/water, 60:12:12 (v/v) and A: MeOH/*tert*-butyl-methyl ether, 4:1 (v/v)]. The column was developed at a flow rate of 0.6 ml min^-1^ with a linear gradient from 50% B to 40% B within 20 min, then to 0% B within 5 min, maintaining the final conditions for another 5 min. Phytoene standard mixture was obtained as described (Schaub et al., 2012).

### Generation of Constructs for RNAi and Root Transformation of *M. truncatula*

Based on the binary vector system of Rene Geurts (RedRoot, Limpens et al., 2004) a new vector system was developed compatible with the Golden Gate cloning system for facile cloning. The spacer was replaced by the *Arabidopsis thaliana* phytoene desaturase (*PDS*, At4g14210) intron in construct pAGH11978 to generate the RNAi vector pAGT1199, in which a 230 bp DNA fragment from the C-terminus and 3′-UTR of MtPSY3 is expressed under the control of the 35S promoter in sense and antisense direction separated by the intron spacer (Supplementary Figure S1). The constructs were transformed into *Agrobacterium rhizogenes* strain Arqua-1 by electroporation as described (Floss et al., 2008a). In parallel, seeds of *M. truncatula* were germinated on 0.7% plant agar for 48 h in the dark at 12°C. The seed coat was removed and 3 mm of the root tip was cut off. The cut-off root was dipped into the bacterial lawn and transferred to square plates with Fahraeus medium kept vertically (Supplementary Figure S1). Plates were incubated under ambient conditions in a climate chamber (16 h light, 90 μM light intensity, 20°C day/17°C night) for 1 week. Subsequently, they were transferred to a regular chamber with 24°C day/20°C night temperature. Fluorescent transformed roots were selected 3–4 weeks after transformation by cutting of non-transformed roots under a fluorescence stereo microscope (LEICA MZIII) as described (Floss et al., 2008a). Plantlets were then cultivated for 1 week on expanded clay. After another round of selection roots were exposed to the various conditions as indicated for 42–49 days prior to harvest. RNA extraction and metabolite analyses were performed from fluorescing roots.

### Determination of Carotenoids and Apocarotenoids

For phytoene quantification from roots, a protocol from Fester et al. (2002b) was adapted. A total of 25 mg of lyophilized root material (250 mg fresh weight) of *M. truncatula* was extracted by vortexing with 400 μl MeOH. After addition of 400 μl of high salt buffer (50 mM Tris/HCl pH 7.5, 1.5 M NaCl), and mixing CHCl_3_ (1 ml) was added. After centrifugation the lower organic phase was removed into a new tube and dried under nitrogen. The pellet was resuspended in 150 μl ethyl acetate, of which 20 μl were injected for analysis. Chromatographic separation of phytoene was achieved by UPLC (Acquity, Waters) on a PRONTOSIL C30 column (Bischoff, Leonberg, Germany; 250 mm × 2 mm × 3 μm) which was kept at 22°C. A binary gradient of A: 5 mM ammonium formate in MeOH and B: MTBE was applied: 0 min: 0% B; 15 min: 57% B; 21 min: 82% B; 22.5 min: 100% B; 26 min: 100% B; 27 min: 0% B; 30 min: 0% B with 600 μl min^-1^ throughout. Samples were cooled to 4°C and full loop injections of 20 μl were conducted. MS acquisition was performed on a QTRAP6500 (Sciex, Toronto, Canada) using a Dual-Source in APCI positive mode. The source parameters were as follows: CAD gas: -2 (arbitrary units), curtain gas (CUR):40 psi, GS1: 60 psi, GS2: 70 psi, nebulizer current: 3 mA, source temperature: 450°C. Phytoene was quantified in MRM mode using these transitions: Q1/Q3: 545.5/81.0 and Q1/Q3: 545.5/69.0 and the following parameters: EP: 10 V; DP: 60 V; collision energy (CE): 40 V; DXP: 15 V. The MS/MS fragmentation pattern of phytoene was verified by enhanced product ion spectra using the same parameters but a CE of 55 V and CE spread of 45 V. The ion trap accumulation time was set to 800 ms. Phytoene was verified by comigration and MS/MS comparison with reference compounds.

SL extraction and quantification from hairy roots of *M. truncatula* was performed by comparing retention time and mass transitions in extracts from equal amounts of root material (250 mg) with those of an available didehydro-orobanchol standard using ultra performance LC coupled to MS/MS using [^2^H6]2′-epi-5-deoxystrigol as an internal standard, as previously described (van Zeijl et al., 2015).

C_13_ α-ionol and C_14_ mycorradicin derivatives were quantified from 200 mg root samples as described (Floss et al., 2008b). Information on the structures of various C_13_ α-ionol (cyclohexenone) glycosides and their relative abundance as well as information on C_14_ mycorradicin derivatives can be found in Schliemann et al. (2008) and in references cited therein.

### Phylogenetic Analysis

Molecular phylogenetic analyses were performed using a Bayesian approach (Huelsenbeck et al., 2001) or by an ML approach (Tamura et al., 2013). For the Bayesian inference of phylogeny the MrBayes plugin in the Geneious^®^ software package (version 3.2.6) was used with default settings (Huelsenbeck and Ronquist, 2001). Multiple sequence alignments of amino acid sequences were done in Geneious^®^ using CLUSTALW algorithm and default settings unless indicated otherwise. For ML analyses alignments were exported in the MEGA format for phylogenetic analysis by the ML method in MEGA6 (Tamura et al., 2013). The bootstrap consensus tree inferred from 1000 replicates was taken to represent the evolutionary history of the taxa analyzed (Felsenstein, 1985). Positions containing gaps, missing data and regions not conserved were eliminated.

### Databases, Software, and Statistics

Nucleotide and amino acid sequence analysis was done with the Geneious^®^ software package version R7^1^. The Medicago Gene Expression Atlas version 3.0 (MtGEA; He et al., 2009) was used through its web server^2^. Entries for target genes were identified by BLAST searches^3^ or by searches for coexpressed genes. Tomato sequence information was obtained through the Solgenomics network^4^. Legume sequence information was obtained from https://legumeinfo.org/. Most amino acid sequences were retrieved from the Phytozome Plant Comparative Genomics Portal Release 11.0^5^.

For statistical treatments datasets were compared using Student’s *t*-test or by analysis of variance (ANOVA) followed by *post hoc* Tukey HSD calculations. Bootstrapping for the phylogeny analysis was done by the MEGA6 software.

### Accession Numbers

Sequence data from this article can be found in the EMBL/GenBank, Phytozome, MtGEA, and other data libraries under the accession numbers or IDs listed in Supplementary Table S1.

## Results

### Identification of AM- and Phosphate Starvation-Inducible *PSY* Isogenes in *M. truncatula* and *S. lycopersicum*

*Medicago truncatula* is a model legume, which offers a wealth of resources and tools for genomic and transcriptomic studies including a gene expression atlas (MtGEA see text footnote 2). Since the *PSY* gene family of this legume has not been analyzed, we first searched the sequenced genome of *M. truncatula* for *PSY* and *PSY*-like sequences via the Phytozome web server (see text footnote 5) using the single copy AtPSY amino acid sequence as a query. We identified four gene models (intact ORFs) for *bona fide* PSYs, which were named MtPSY1, MtPSY2a, MtPSY2b, and MtPSY3 (**Table 1**). MtPSY3 is more distantly related to AtPSY but is highly similar to the previously identified cassava MePSY3 (70% identity) and other dicot PSYs previously annotated as PSY3. However, MtPSY3 is only quite distantly related to rice PSY3 (48% identity). We first checked expression profiles of the corresponding genes in the MtGEA by selecting 22 samples out of the roughly 250 organ, tissue and treatment datasets currently available. *MtPSY1* showed highest transcript levels in leaves and other photosynthetic tissues, moderate to lower levels in most other tissues and low levels in most of the root genotypes and conditions. *MtPSY3* exhibited a very different expression profile exhibiting strong expression only upon root colonization by AM fungi (Supplementary Figure S2). Moderately elevated transcript levels can be detected in phosphate-starved roots, particularly upon strong Pi deprivation (supply reduced from 2 mM to 20 μM, sample #16 vs. #18) and also in roots accommodating symbiotic nodule-forming rhizobacteria (*Sinorhizobium meliloti*, sample #10) (Supplementary Figure S2). A comprehensive dataset is shown in Supplementary Figure S3. *MtPSY2a/MtPSY2b* were not reliably covered in the MtGEA.

**Table 1:** Designations and features of *PSY* isogenes from *M. truncatula* (*Mt*) and *S. lycopersicum* (*Sl*).

Designation	Genome ID	MtGEA ID	Deduced protein including transit peptide (aa)	Amino acid sequence identity to AtPSY (%)
*MtPSY1*	Medtr5g076620	Mtr.12722.1.S1_at	434	67.3
*MtPSY2a*	Medtr3g450510	–	388	67.7
*MtPSY2b*	Medtr5g090780	–	395	67.7
*MtPSY3*	Medtr3g083630	Mtr.45966.1.S1_at	387	54.3
*SlPSY1*	Solyc03g031860	–	412	68.2
*SlPSY2*	Solyc02g081330	–	437	69.2
*SlPSY3*	Solyc01g005940	–	384	53.1

*Genome IDs and MtGEA IDs were retrieved from the Phytozome or MtGEA web servers, respectively. Deduced protein length (amino acids, aa) including transit peptides were taken from gene models. Amino acid sequence identities were calculated be Geneious using default settings.*

**FIGURE 2:**
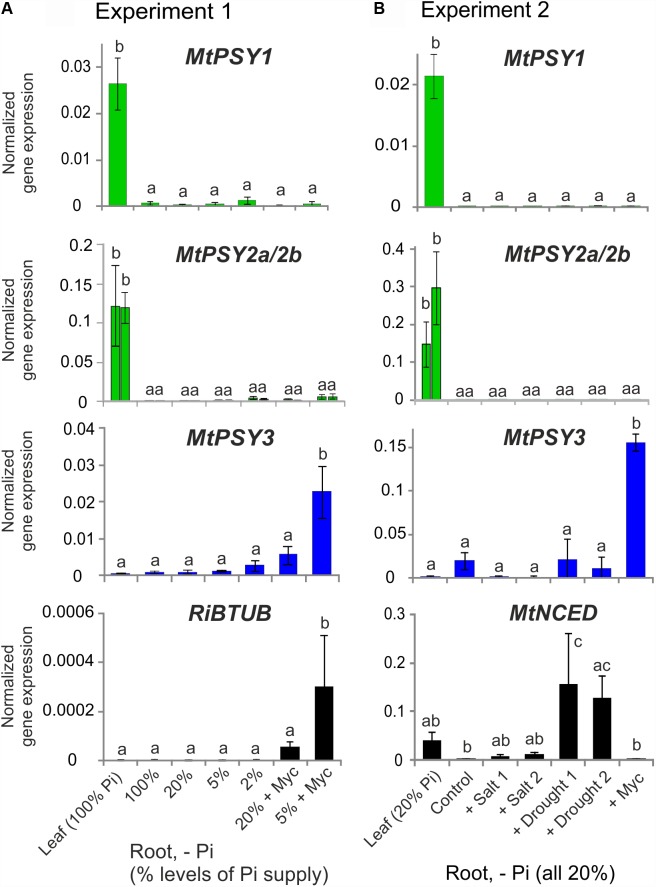
Normalized transcript levels of *PSY* isogenes from *M. truncatula* under phosphate-starvation, mycorrhization, salt and drought stress conditions in leaves and roots. Plants from two independent experiments **(A,B)** were harvested after 5 weeks of cultivation in expanded clay under the various phosphate starvation (– Pi), mycorrhization (+ Myc) salt treatment (+ salt) or drought (+ drought) conditions as indicated. qRT-PCR data were normalized to constitutively expressed *MtEF1α*. Error bars indicate SD from at least three biological replicates. An AM fungal marker gene (*R. irregularis β-TUBULIN*, *RiBTUB*) or a marker gene of ABA biosynthesis (*MtNCED*) were included in the analysis of experiment 1 or 2, respectively (lower panels). Please note adapted scales in the various panels. Error bars derive from SD of at least three biological replicates. Different lower case letters indicate statistically different values in ANOVA followed by a Tukey HSD test (*p* ≤ 0.05).

These results on *M. truncatula* were validated and extended by performing qRT-PCR analyses for leaves and for roots exposed to various conditions of phosphate (Pi) starvation alone or together with mycorrhization as well as to drought and salt stress. We used an AM fungal gene (*R. irregularis β-TUBULIN*, *RiBTUB*) and a single copy *MtNCED* gene (Medtr2g070460) as molecular markers to ascertain that the plants were mycorrhized or were responding to the abiotic stress treatments toward ABA biosynthesis, respectively. *MtPSY1* was confirmed to be strongly expressed in leaves but only weakly in all root conditions (**Figure 2**). Both *MtPSY2a* and *MtPSY2b* exhibited expression profiles similar to *MtPSY1* but quantitatively their transcript levels in leaves were higher than *MtPSY1*. *MtPSY3* expression in leaves was extremely low, but was detectable in roots already at full Pi supply (100%). Under strong Pi deprivation (2% of normal Pi supply) *MtPSY3* did show a trend toward higher transcript levels but this did not turn out to be statistically significant (**Figure 2A**). Mycorrhization under Pi deprivation was confirmed to be the only condition for strongly and significantly elevated transcript levels of the *MtPSY3* gene (**Figure 2**). Its expression strength correlated well with the level of fungal root colonization as indicated by the *RiBTUB* marker gene and was always markedly higher in mycorrhizal roots compared to non-mycorrhizal roots at the same level of Pi deprivation (**Figure 2A**). *MtPSY3* responsiveness to salt stress and drought under conditions of moderate Pi deprivation (20% Pi) was absent and even decreased upon salt treatment (**Figure 2B**). The *MtNCED* marker gene was induced strongly only by the two drought treatments, but not by mycorrhization (**Figure 2B**).

**FIGURE 3:**
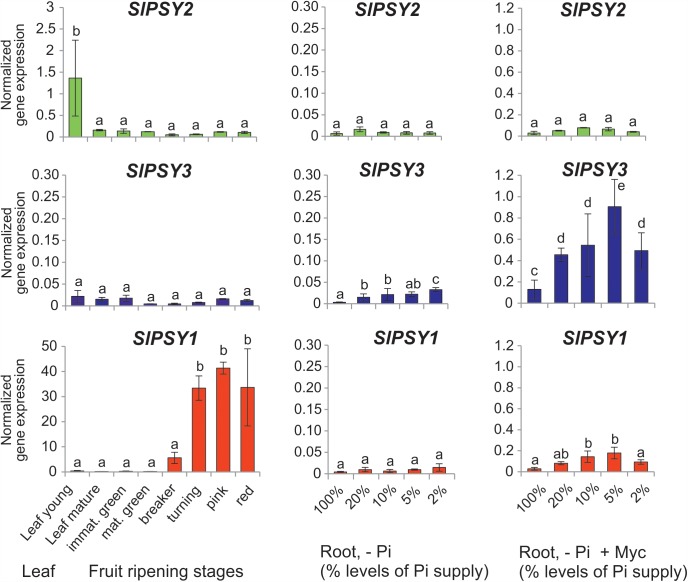
Normalized transcript levels of *PSY* isogenes from *S. lycopersicum* in phosphate-starved roots with and without mycorrhization and in other organs. Roots were harvested after 6 weeks of cultivation on expanded clay under phosphate starvation (– Pi, center panels) or mycorrhizal conditions under various phosphate starvation regimes (– Pi + Myc, right panels). Leaf and ripening fruit samples (left panels) were taken from soil-grown plants. The housekeeping gene *SlPSY2* is presented on the top panels (green columns). *SlPSY3* as the putative ortholog of *MtPSY3* is presented in the center (blue columns). The special paralog of tomato (*SlPSY1*) is shown on the bottom (red columns). Please note the individual adaptation of scales in the various data displays due to very high differences in transcript levels between the three *PSYs* and the various organs and treatments. However, the same scale has been maintained for the three genes in the – Pi and – P + Myc treatments of roots for better comparison. qRT-PCR data were normalized to constitutively expressed *SlEF1α*. Error bars indicate SD from three to five biological replicates for the root samples and two replicates for the leaf and fruit samples. Different lower case letters indicate statistically different values in ANOVA followed by a Tukey HSD test (*p* ≤ 0.05).

Since tomato has been the prototype system for plant *PSY* gene functional analyses we next searched for mycorrhization and phosphate starvation-responsive *PSY* isogenes in this species. In addition to the *SlPSY1* and *SlPSY2* genes introduced above a third gene (*SlPSY3*) was described earlier in tomato and is contained in the genome sequence (**Table 1**). *SlPSY3* was shown to have low but detectable transcript levels in roots, whereas these were very low to undetectable in all other organs and tissues (Fantini et al., 2013). We set up mycorrhization and phosphate starvation experiments for root analyses but also included two stages of leaf development and six stages of fruit ripening in qRT-PCR analyses of the three tomato *PSY* genes. As expected, the housekeeping *SlPSY2* had high transcript levels in young leaves, moderate transcript levels in fruits and very low levels in all of the root conditions tested (**Figure 3**). *SlPSY3* had very low levels in leaves and fruits, but exhibited a slight but significant elevation of transcript levels in roots upon increasing deprivation of phosphate down to 2% residual phosphate supply. Mycorrhization of roots cultivated at various levels of phosphate supply resulted in a very strong and significant increase in *SlPSY3* transcript levels reaching a maximum at phosphate supply reduced to 5%. *SlPSY1* exhibited extremely high transcript levels in fruits at late stages of fruit ripening exceeding the highest levels of other *SlPSY* genes by a factor of 40 but also showed a slight elevation in some mycorrhizal samples yet at a much lower level than *SlPSY3* (**Figure 3**).

### MtPSY3 and SlPSY3 Are Members of a Novel Dicot-Specific PSY Clade Distinct From the PSY3 Class of the Poaceae

Although originating from different dicot plant families, MtPSY3 and SlPSY3 have highly similar amino acid sequences (70% identity), whereas the identity score to other PSY isoforms in the same species is lower (55–61%). The score of MtPSY3 to the monocot OsPSY3 is even lower (47%). Aligning a total of 81 PSY amino acid sequences of angiosperms, gymnosperms, mosses, algae, and a bacterial PSY ancestor (crtB) revealed for dicot PSY3s a strikingly short N-terminus and a distinctive stretch within the first 144 amino acids compared to other angiosperm PSYs (Supplementary Figure S4).

**FIGURE 4:**
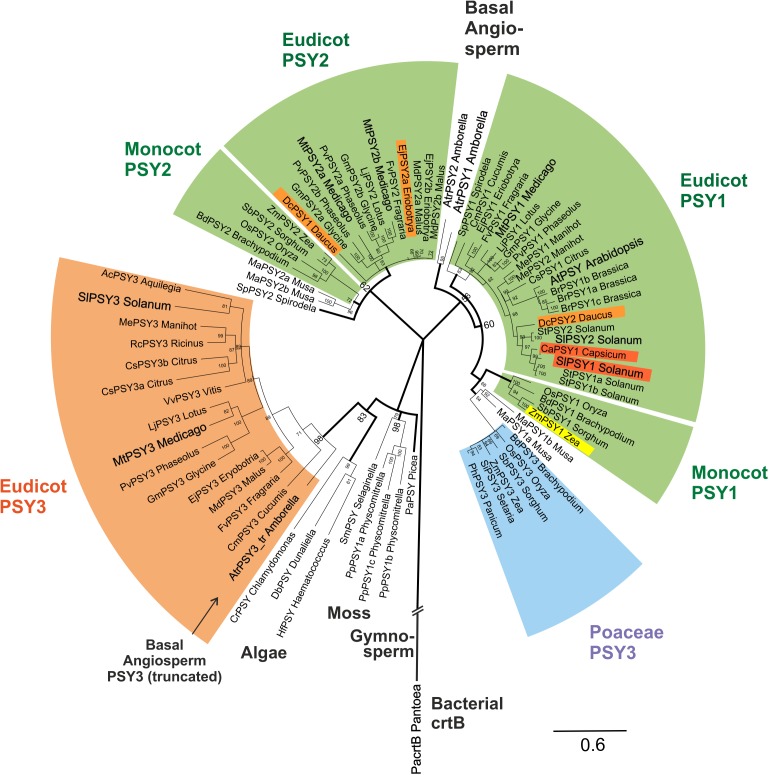
Molecular phylogenetic analysis of PSY amino acid sequences. Eighty one PSY amino acid sequences from bacteria, algae, primitive plants, and higher plants were subjected to a Bayesian inference of phylogeny by the MrBayes plugin of the Geneious software package (Huelsenbeck and Ronquist, 2001). The phylogeny estimation is based on a CLUSTALW alignment shown in Supplementary Figure S4, which included a bacterial crtB sequence and a truncated sequence (192 aa) from the basal angiosperm *A. trichopoda* (AtrPSY3_tr Amborella, marked by arrow). Various clades of angiosperm PSYs are marked by colors and single PSYs investigated in this work are enlarged and in bold text. The newly defined eudicot PSY3 branch (orange clade) is most closely related to PSYs from algae, mosses and a gymnosperm. It clearly separates from the PSY3 of Poaceae (blue clade). Two separate angiosperm clades are assigned mainly to housekeeping photosynthetic functions (green clades, PSY1, PSY2), which also contain variants specifically expressed in single carotenoid-rich organs (colored PSYs within green clades). Posterior probabilities for nodes are given next to the nodes. Accession numbers or gene identifiers are listed in Supplementary Table S1.

This alignment was used for a large-scale comparative phylogenetic analysis of PSYs. The evolutionary history of PSYs was inferred by using a Bayesian approach (Huelsenbeck et al., 2001) applying MrBayes software. MtPSY3 and SlPSY3, the two (eu)dicot PSYs with distinct, strongly AM-inducible expression described in this paper appear in a large, highly conserved clade with 98% bootstrap support, that includes the MePSY3 sequence from cassava (Arango et al., 2010) but not the PSY from Arabidopsis or other Brassicaceae species. We name this clade (eu)dicot PSY3 (dPSY3; **Figure 4**, orange clade). It is clearly distinct from the previously characterized PSY3 clade limited to the Poaceae, which was characterized as being responsive to abiotic stress (**Figure 4**, blue clade).

PSY sequences from several algae, a moss model species (*Physcomitrella patens*) and a gymnosperm (*Picea abies*) are closely related to each other as expected. Surprisingly, the new dPSY3 clade does not cluster among other angiosperm sequences but most closely to these algae, moss, and gymnosperm PSYs classifying this clade as the most ancient one among angiosperm PSYs (**Figure 4**). The “classical” PSYs of angiosperms appear to separate into two major clades. The well-characterized PSYs from tomato (earlier designated SlPSY1, SlPSY2) and Arabidopsis (AtPSY, single copy) but also the MtPSY1 identified in this work fall into one clade, which we call the PSY1 clade (**Figure 4**). From the data available, most genes in this clade (e.g., *MtPSY1*, *SlPSY2*, *MePSY1*, *AtPSY*) are expressed in photosynthetic tissues indicated by the green coloration of this clade. However, there are several exceptions, where members of this clade are also expressed in non-photosynthetic tissues, like *SlPSY1* expressed in fruits. This deviation from expression in green tissues is indicated by separate colorings inside the green clades.

In *M. truncatula*, there are two additional PSYs closely related to each other, which fall into a distinct clade called clade 2 (MtPSY2a, MtPSY2b; **Figure 4**). Clade 2 has many members, which are expressed in photosynthetic tissues characterizing it as another “green” clade (e.g., ZmPSY2, MtPSY2a, MtPSY2b). Similar as in clade 1, there are also some closely related members (paralogs) with additional or preferential expression of their genes in yellow/orange-colored carotenoid-producing organs (EjPSY2a: loquat fruits; DcPSY1: carrot roots).

*Amborella trichopoda* is a single species in a single genus and family, which is viewed as the most basal lineage within so-called basal angiosperms—presumable progenitors of contemporary angiosperms (Soltis et al., 2008). Two *bona fide* PSY sequences closely related to each other were identified from the *A. trichopoda* genome sequence. The phylogenetic analysis places them outside the two green (eu)dicot/monocot clades for which they might have been the progenitors (**Figure 4**). Interestingly, the sequenced genome of this basal angiosperm contains, next to *PSY1* clade-type copies, a truncated gene, whose deduced protein falls into the dPSY3 class. The available sequence covers a stretch of 192 amino acids, which displays 71% identity to MtPSY3 (sequence #77; Supplementary Figure S4) affiliating it to the (eu)dicot PSY3 clade (**Figure 4**). This finding argues for the presence of d*PSY3* genes in ancient genomes before the separation of angiosperms into monocots and dicots. We also performed a PSY phylogeny estimation by the classical maximum likelihood method followed by generating a bootstrap consensus tree through MEGA6 software (Tamura et al., 2013). While there are some minor differences in the two types of phylogenetic analyses (Bayesian vs. maximum likelihood) the use of the second approach confirms the distinct protein structure and ancient evolutionary origin of the new dPSY3 clade (99% bootstrap support; Supplementary Figure S5).

To prove functionality of a dPSY3 clade member we selected SlPSY3 for *in vitro* assays of recombinant protein. 6^∗^His-SlPSY3 and 6^∗^His-ZmPSY1 were purified following an established protein refolding procedure revealing monodisperse (aggregate-free) proteins (Supplementary Figure S6). 6^∗^His-SlPSY3 was incubated together with recombinant, purified 6^∗^His-GGPP synthase 11 from Arabidopsis in the presence of DMAPP and IPP. Only when recombinant SlPSY3 and AtGGPPS11 were combined, the assay resulted in the production of a compound, which had properties identical to the compound from the positive control assay with ZmPSY1 (Supplementary Figure S7A). The identity of the compound produced by SlPSY3 and ZmPSY1 as 15,15′-*cis*-phytoene was further demonstrated by comparison with a 15,15′-*cis/trans*-phytoene mixture obtained from bacterial enzymes (CrtE and CrtB, corresponding to GGPPS and PSY, respectively; Supplementary Figure S7B). These data confirm that SlPSY3 is a genuine PSY.

### Knockdown of *MtPSY3* Expression Strongly Reduces Strigolactone and α-Ionol Mycorradicin Levels *in Planta*

To investigate the role of MtPSY3 *in planta* we aimed at reducing the levels of *MtPSY3* transcripts in mycorrhizal and non-mycorrhizal roots by a transgenic approach. We selected a *M. truncatula* whole plant/transgenic hairy root system, in which the shoot remains in a non-transformed state. A 230 bp DNA fragment from *MtPSY3* covering the region of the C-terminus of the protein from amino acid position 394 downward and extending 41 bp into the 3′-untranslated sequence was used for RNAi experiments. The nucleotide sequence identity in this region between *MtPSY3* and *MtPSY1/MtPSY2a* is only 53%/62%, respectively.

**FIGURE 5:**
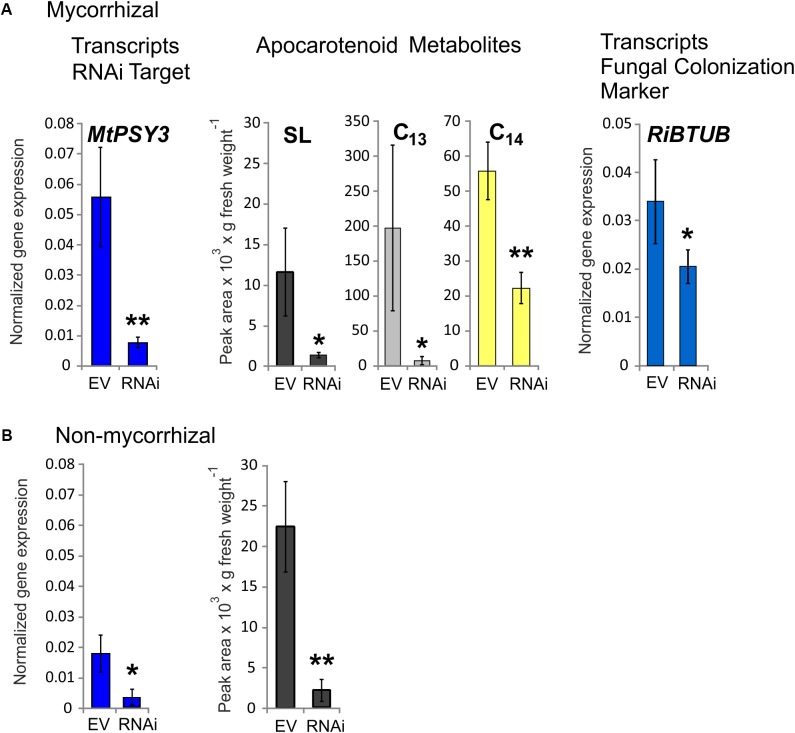
Knockdown by RNAi of *MtPSY3* expression in transgenic hairy roots of *M. truncatula* affecting apocarotenoid levels and fungal root colonization. **(A)** Transgenic roots of mycorrhizal empty vector (EV) control plants compared to *MtPSY3*-RNAi-suppressed transgenic roots. EV and RNAi, *n* = 3 each. **(B)** Transgenic roots of non-mycorrhizal EV and *MtPSY*3-RNAi suppressed plants. EV, *n* = 3 + one unsuppressed RNAi plant; RNAi, *n* = 2. All plants were cultivated with 20% Pi supply. Root fractions were analyzed for *MtPSY3* transcript levels by qRT-PCR (left panels, blue columns). In mycorrhizal samples, a fungal marker gene for root colonization (*RiBTUB*) was included in the analysis (right panel in **A**). Data were normalized to constitutively expressed *MtEF1α*. In additional fractions various root apocarotenoid derivatives (SL, C_13_ α-ionols and C_14_ mycorradicin as indicated) were quantified from mycorrhizal roots and SL was determined also from non-mycorrhizal roots (center panels in **A** or right panel in **B**). Mean values ± SD are shown. RNAi data were compared with EV controls by Student’s *t*-test (^∗^*p* ≤ 0.05, ^∗∗^*p* ≤ 0.01).

Three RNAi plants from a series of mycorrhized transformants, which had the lowest *MtPSY3* transcript levels in roots were selected and compared to three mycorrhizal empty vector (EV) control plants. Average *MtPSY3* transcripts of these RNAi plants were reduced to about 10% residual levels relative to EV controls (**Figure 5A**). Levels of didehydro-orobanchol, the major SL derivative in *M. truncatula*, were strongly reduced in roots of the RNAi plants to about the same extent (10% relative to controls) as the *MtPSY3* transcripts (**Figure 5A**). We further determined the levels of C_13_ α-ionol glycosides and C_14_ mycorradicin in these samples by again selecting the most abundant structural variant of either of these classes. C_13_ α-ionol levels were very strongly reduced in the roots of the RNAi plants to only about 5% of EV controls, whereas C_14_ mycorradicin was also significantly reduced but only to about 40% of controls. These results clearly show a strong correlative effect of *MtPSY3* transcript downregulation on the levels of three types of symbiosis-supporting root apocarotenoids, namely SL (didehydro-orobanchol), C_13_ α-ionol glycosides, and C_14_ mycorradicin in mycorrhizal roots. As SLs are known to promote root colonization by AM fungi we next determined the levels of a fungal marker gene transcript (*RiBTUB*) for root colonization in mycorrhizal *MtPSY3*-RNAi roots vs. mycorrhizal EV roots. The transcript levels of *RiBTUB* were significantly reduced in the RNAi plants (**Figure 5A**). The reduction was not as pronounced as the reduction of *MtPSY3* transcripts and of SL levels, which is in agreement with the view that SLs are not an absolute requirement for root colonization but only a promoting factor.

We also analyzed *MtPSY3*-RNAi and EV transformants, which were not exposed to AM fungi. In the non-mycorrhizal experiments we obtained only two individual RNAi plants, which showed strongly downregulated *MtPSY3* transcript levels. We therefore selected a third RNAi plant in the analysis, which was not downregulated and included its data with three non-mycorrhizal EV plants jointly used as controls. *MtPSY3* transcripts in the two downregulated RNAi plants were reduced to about 17% of control levels (**Figure 5B**). Levels of the SL derivative didehydro-orobanchol were reduced in the two non-mycorrhizal RNAi plants to a similar extent (about 10% residual SL relative to controls; **Figure 5B**). We also checked transcript levels of other *MtPSY* isogenes (*MtPSY1*, *MtPSY2a*) in both mycorrhizal and non-mycorrhizal samples. There were no significant alterations in either *MtPSY1* or *MtPSY2a* in the RNAi plants relative to EV controls (Supplementary Figure S8).

### Chemical Inhibition of Metabolite Flux Reveals Strongly Elevated Carotenoid (Phytoene) Levels Upon Mycorrhization

**FIGURE 6:**
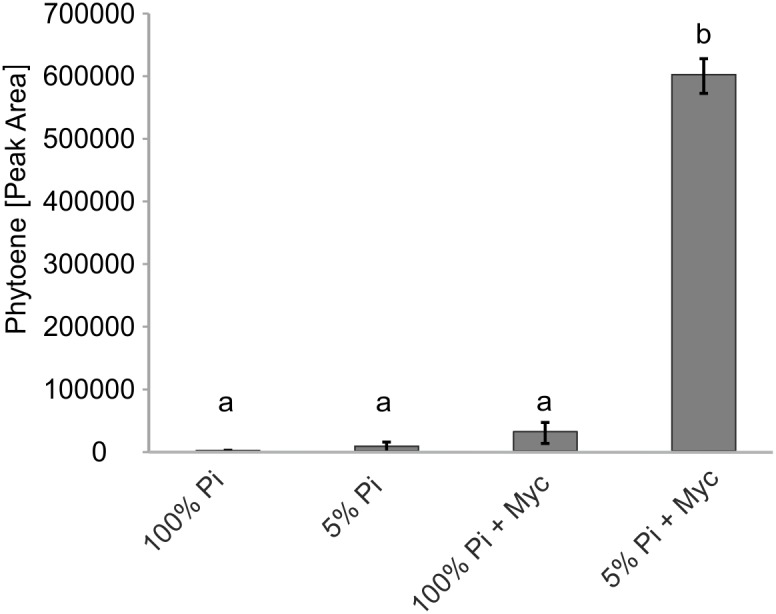
Impact of Pi starvation and mycorrhization on phytoene levels after norflurazon treatments. *M. truncatula* plants grown under two phosphate supply (100% Pi, 5% Pi) conditions with or without mycorrhization (Myc) as indicated were irrigated with a norflurazon solution for 8 days before harvest after 6 weeks of cultivation. Root extracts were analyzed for relative phytoene content by LC-MS. Error bars derive from SD of three biological replicates. Different lower case letters indicate statistically different values in ANOVA followed by a Tukey HSD test (*p* ≤ 0.01).

Transcriptional control is but one of many levels of control on metabolic pathways. We therefore aimed to demonstrate activation of carotenogenesis in AM-colonized roots also at the metabolite level. The chemical inhibitor norflurazon can specifically block the enzyme activity of phytoene desaturase (PDS, reaction step subsequent to PSY) leading to an accumulation of its substrate phytoene. Further pathway flux into downstream carotenoids and apocarotenoids is thus effectively prevented, as already shown for SL production (Jamil et al., 2010). Without norflurazon phytoene was not detectable in mycorrhizal roots presumably because this intermediate is quickly metabolized (Fester et al., 2002b, 2005). We used *M. truncatula* plants cultivated under some of the specific phosphate supply and mycorrhizal conditions used for the transcript determination experiments. Phytoene was hardly detectable in non-mycorrhizal roots receiving full (100%) phosphate supply (**Figure 6**), but its concentration was about threefold elevated upon strong phosphate starvation (5% of regular supply). Mycorrhization under non-favorable conditions (100% Pi) resulted in a 12-fold increase relative to the non-mycorrhizal status under the same level of Pi supply. However, only strong mycorrhization under the AM-favorable condition of phosphate supply reduced to 5% led to a very strong and significant increase in phytoene levels, i.e., 20-fold relative to the non-favorable mycorrhizal condition and 240-fold relative to the negative control (no mycorrhiza, full phosphate supply; **Figure 6**).

### Additional Isogenes of Carotenogenesis Are Responsive to Mycorrhization and Are Coregulated With d*PSY3* and Downstream Carotenoid Cleavage Steps

**FIGURE 7:**
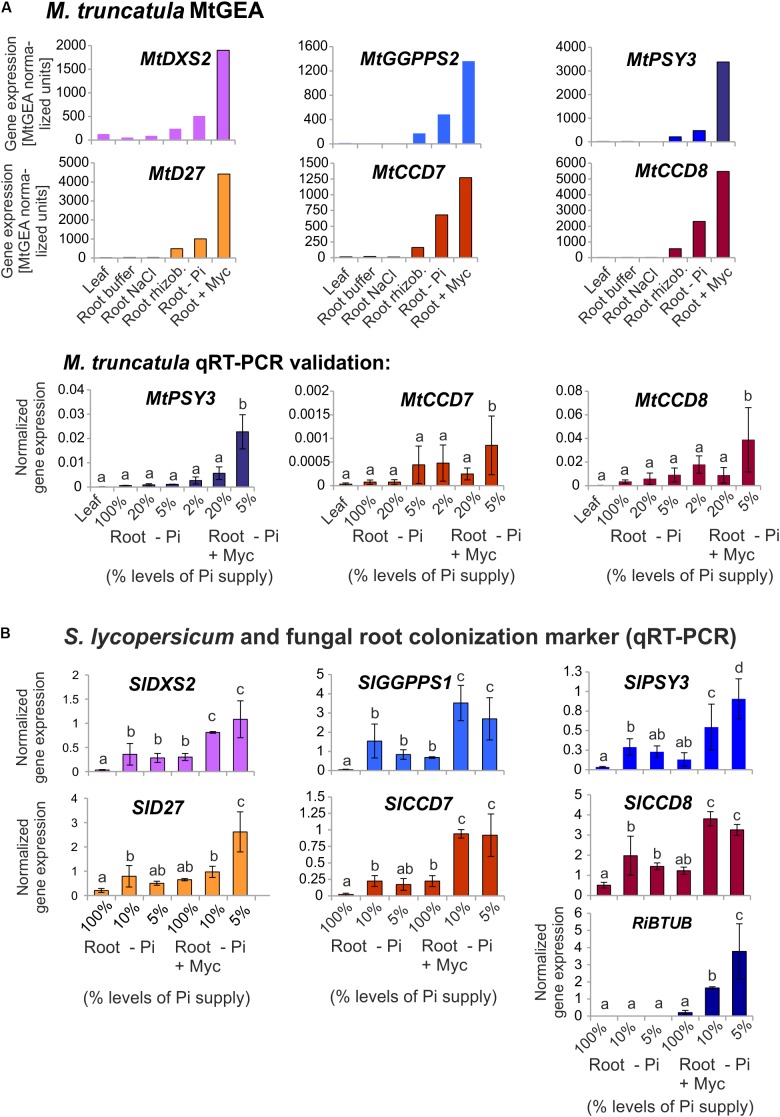
Coexpression analysis of *MtPSY3* and *SlPSY3* with isogenes in upstream and downstream steps. **(A)** *M. truncatula* gene transcript data for candidate genes retrieved from MtGEA using a limited probeset (# 1, 9, 10, 15, 18, 21) out of 22 used in the analysis in Supplementary Figure S2. Target genes were *DXS2*, *GGPPS2* for upstream and *D27*, *CCD7*, and *CCD8* for downstream steps toward SL biosynthesis. MtGEA data for *MtCCD7* and *MtCCD8* were validated for root treatments by qRT-PCR experiments. qRT-PCR data were normalized to constitutively expressed *MtEF1α*. **(B)** *S. lycopersicum* samples were analyzed by qRT-PCR using a simplified set of samples. The AM-responsive *GGPPS* is termed here *SlGGPPS1* for historical reasons. *MtPSY3* and *SlPSY3* data from **Figures 2** and **3** are included for comparison. In addition, transcript levels of the fungal *RiBTUB* gene were determined. Data were normalized to constitutively expressed *SlEF1α*. Error bars in qRT-PCR data are derived from SD of four biological replicates. Please note different scales in the various data displays. Error bars derive from SD of two to five biological replicates. Different lower case letters indicate statistically different values in ANOVA followed by a Tukey HSD test (*p* ≤ 0.05).

Using the MtGEA electronic coexpression analysis tool on the full dataset of *MtPSY3* expression as a query resulted in the identification of a *GGPP SYNTHASE* isogene (Medtr5g019460, termed *MtGGPPS2*), whose encoded protein catalyzes substrate formation for PSY. Moreover, previous experiments have identified an AM-regulated isogene further upstream of *PSY* and *GGPPS* in the MEP pathway (*MtDXS2*, Walter et al., 2002; Floss et al., 2008a). For an *in silico* coexpression analysis using MtGEA data, we further selected three downstream steps of apocarotenoid biogenesis encoding β-carotene isomerase (D27) and the two carotenoid cleavage steps (CCD7, CCD8) in the biosynthetic pathway to SLs (see **Figure 1** for positions in the pathway and **Table 2** for identifiers of AM-responsive and non-responsive isogenes). We retrieved selected MtGEA data from a leaf sample and five root conditions including phosphate starvation and mycorrhizal colonization for a simplified comparative analysis. Resembling the *MtPSY3* expression profile, all genes showed strong transcriptional responsiveness to the mycorrhizal condition and a moderate responsiveness to phosphate starvation in roots, whereas there were very low or undetectable transcript levels in leaves (**Figure 7A**, top panels). The results were validated by qRT-PCR for *MtCCD7* and *MtCCD8* on the same RNA samples that were used already for the analysis presented in **Figure 2** (leaf, phosphate starvation series on roots without and with AM fungal colonization). The qRT-PCR results confirm the strong AM-responsiveness of these two genes (**Figure 7A**, lower panels).

**Table 2:** Gene families with AM-responsive isogenes in carotenogenesis and downstream carotenoid cleavage steps coregulated with *PSY3* from *M. truncatula* (*Mt*) and *S. lycopersicum* (*Sl*).

*M. truncatula*			*S. lycopersicum*	
Designation	Genome ID	MtGEA ID	Designation	Genome ID
*MtDXS1*	Medtr8g078071	Mtr.48548.1.S1_at	*SlDXS1*	Solyc01g067890
***MtDXS2***	**Medtr8g068265**	**Mtr.43585.1.S1_at**	***SlDXS2***	**Solyc11g010850**
*MtGGPPS1*	Medtr8g078070	Mtr.46184.1.S1_at	***SlGGPPS1***	**Solyc11g011240**
***MtGGPPS2***	**Medtr5g019460**	**Mtr.31291.1.S1_at**	*SlGGPPS2*	Solyc04g079960
***MtD27***	**Medtr1g471050**	**Mtr.11343.1.S1_at**	***SlD27***	**Solyc09g065750**
*MtD27b*	Medtr7g095920	Mtr.28056.1.S1_at	*-*	-
***MtCCD7***	**Medtr7g045370**	**Mtr.32038.1.S1_at**	***SlCCD7***	**Solyc01g090660**
***MtCCD8***	**Medtr3g109610**	**Mtr.37123.1.S1_s_at**	***SlCCD8***	**Solyc08g066650**
*MtCCD8b*	Medtr7g063800	Mtr.1606.1.S1_at		

*Genome IDs and MtGEA IDs were retrieved from Phytozome and MtGEA web servers, respectively. AM-responsive gene family members are indicated in bold.*

We next analyzed by qRT-PCR tomato (iso)genes coding for the five steps found above in *M. truncatula* to have preferentially AM-responsive (iso)genes for potential coregulation with *SlPSY3* (summarized in **Table 2**). Among two putative *SlGGPPS* isogenes we found the gene called *SlGGPPS1* to be responsive to mycorrhization (**Figure 7B**). This designation has been established earlier (Ament et al., 2006) and is maintained here as is the designation of *SlGGPPS2* investigated in the same study. The *SlD27* gene candidate Solyc09g065750 was also included. A single copy-*SlCCD7* gene has been shown to be expressed in roots and green fruits (Vogel et al., 2010) but also to be responsive to mycorrhization of roots (López-Ráez et al., 2015). Both Pi-starvation and AM-responsiveness was observed in our experiments (**Figure 7B**). The single copy-*SlCCD8* has been shown to be highly expressed in roots (Kohlen et al., 2012). It was shown to be responsive to phosphate starvation but not further responsive to mycorrhization under particular conditions applied (López-Ráez et al., 2015). We confirm here the phosphate-starvation responsiveness of *SlCCD8* and also found a further elevation in mycorrhizal roots yet only under strong phosphate starvation conditions (**Figure 7B**). Fungal *RiBTUB* transcripts in tomato roots were below detection level in non-mycorrhizal controls but correlated well with the alterations of *SlPSY3* transcripts in the various mycorrhizal root samples (**Figure 7B**) in a manner comparable to *MtPSY3* (see **Figure 2**).

## Discussion

### Dicot-PSY3 Is Primarily Committed to Production of Symbiosis-Supporting Strigolactone Apocarotenoids

We have described here a widespread new clade of PSY genes and proteins (bootstrap support 98–99%), which is restricted to dicots. Based on existing annotations we propose to name this clade (eu)dicot PSY3 (dPSY3) to distinguish it from the PSY3 of the Poaceae to which it is phylogenetically distinct (**Figure 4** and Supplementary Figure S5). d*PSY3*-type genes were recognized previously in several studies (Arango et al., 2010; Peng et al., 2013; Fu et al., 2014; Ampomah-Dwamena et al., 2015) but no major developmental or environmental stimulus for their transcriptional activation was reported thus far except for low expression confined to roots in melon (Qin et al., 2011) and tomato (Fantini et al., 2013). The report of root colonization by AM fungi as a single strong cue for transcriptional activation of d*PSY3* genes is thus one major novelty of this paper. This is shown for two d*PSY3s* from distantly related plant lineages [legumes, *MtPSY3* (**Figure 2**) and Solanaceae, *SlPSY3* (**Figure 3**)] but may extend to members from many other dicot families. This idea is in line with a bioinformatics-based finding that *MtPSY3* is an AM-responsive gene highly conserved among plants (Favre et al., 2014). The extent of d*PSY3* transcript elevation in mycorrhizal roots was quite variable but always coincided with the strength of root colonization, which itself correlates with the extent of phosphate deprivation (**Figures 2**, **3**, **7**). At all levels of phosphate supply d*PSY3* transcript levels were much higher in mycorrhizal compared to non-mycorrhizal roots suggesting that the presence of the fungi constitutes the strongest cue and not phosphate starvation, which is actually expected to be alleviated upon the presence of the symbionts.

There are some striking arguments that the main symbiosis-supporting molecules produced through the activation of AM-induced d*PSY3* genes are the SLs. Both *MtPSY3* and *SlPSY3* are concomitantly upregulated with downstream biosynthetic steps leading to SLs, namely carotenoid isomerization (D27) and the two subsequent carotenoid cleavage steps (CCD7, CCD8) (**Figure 7**). While CCD7 is also involved in the production of other root apocarotenoids (C_13_ α-ionols and C_14_ mycorradicin, see below), CCD8 is committed to SL biogenesis. The coregulation of *MtPSY3* and *SlPSY3* with their respective *CCD8* genes argues in favor of dPSY3 acting mainly as a provider of precursor molecules toward the production of SLs. This view is in agreement with the results of knockdown experiments of *MtPSY3* expression in transgenic hairy roots of *M. truncatula*. A decrease in *MtPSY3* transcript levels in roots strictly coincided with the extent of reduction of the SL didehydro-orobanchol in both a mycorrhizal and a non-mycorrhizal condition (**Figure 5**). This results also leaves very little, if any, contribution of other *PSY* isogenes to SL production, some of which are slightly upregulated upon mycorrhization, but whose expression is much lower than *MtPSY3*.

The timing of d*PSY3* and coregulated *CCD8* expression in mycorrhizal roots being strong in late stages of the symbiosis suggests a continuous need for SL production in all and also the later stages of the symbiosis. Contrary to popular belief, SLs thus appear to be needed for a sustained support of AM fungi and the symbiosis and not only for symbiont attraction or early encounters of symbionts and mutual exchanges of plant (SL) and fungal (Myc factor) signals. Furthermore, comparing the data on symbiosis-supporting SL production indicators vs. root-extractable levels of SLs in mycorrhizal roots reveals a paradox. Both the combined transcript results on genes of (apo)carotenogenesis (**Figure 7**) and the norflurazon inhibitor studies (**Figure 6**) imply an elevated and sustained production of SLs and their precursors during mycorrhization. On the other hand we have observed in EV control plants alterations of extractable SL levels in mycorrhizal roots relative to non-mycorrhizal controls in the opposite direction, i.e., the levels are reduced (**Figure 5A** vs. **Figure 5B**). The latter is not a new observation but has been reported repeatedly by researchers working on the stimulatory effects of SLs on seed germination of parasitic weeds from the Orobanchaceae (Cardoso et al., 2011; López-Ráez et al., 2011a). In this context, mycorrhization has even been suggested as a strategy for parasitic weed management reducing seed germination of weeds through reduced SL exudation. Collectively, these observations thus argue for an AM-induced production of SLs by the plant but accompanied by SL metabolization/catabolism presumably brought about by the fungus resulting in reduced extractable or exuded levels of SLs (Koltai, 2014; Walter et al., 2015). The drain on SL from such fungal activities or simply from exuding SLs into the rhizosphere may necessitate to maintain a continuously high level of SL production in roots in both presymbiotic and symbiotic stages.

A closer look into the MtGEA data reveals elevated *MtPSY3* transcripts also in another root symbiosis, namely the rhizobium/root interaction supporting nitrogen acquisition (**Figure 7A** and Supplementary Figure S2). Interestingly, the expression of a *MtD27* gene has recently been shown to be co-opted in the rhizobium symbiosis being highly expressed locally in nodule primordial and infection zones (van Zeijl et al., 2015). Moreover, both *MtD27* and *MtCCD8* are significantly upregulated in the root hair infectome after rhizobium inoculation (Breakspear et al., 2014). Both reports argue for a role of SLs in rhizobial infection structure and nodule development.

Levels of C_13_ α-ionol apocarotenoids accumulating in arbusculated cells also followed the levels of residual *MtPSY3* transcripts in the *M. truncatula* knockdown lines, while C_14_ mycorradicin was also significantly reduced albeit only to a more moderate extent (**Figure 5A**). The fate of arbuscules could not be determined in these lines but previous C_13_/C_14_ apocarotenoid suppression through *MtDXS2* knockdown was correlated with a shift in the population structure of arbuscules leading to an increase in old arbuscule individuals at the expense of mature ones (Floss et al., 2008a). These and other observations have led to a model, in which C_13_ apocarotenoids (but not C_14_) participate in control of the arbuscular life cycle by the plant host accelerating turnover of those arbuscules, which are ineffective in symbiotic phosphate transfer (Walter et al., 2010; Walter, 2013). From our experience with the glycosides of C_13_ apocarotenoids over some 15 years, these compounds constitute reliable metabolic markers of arbuscule abundance and may be remnants of arbuscule decay and turnover processes (Fester et al., 2002a; Schliemann et al., 2008; Walter et al., 2010; Walter, 2013).

### Isogene-Controlled Carotenoid Supply Chains Channeled Into Apocarotenoids

**FIGURE 8:**
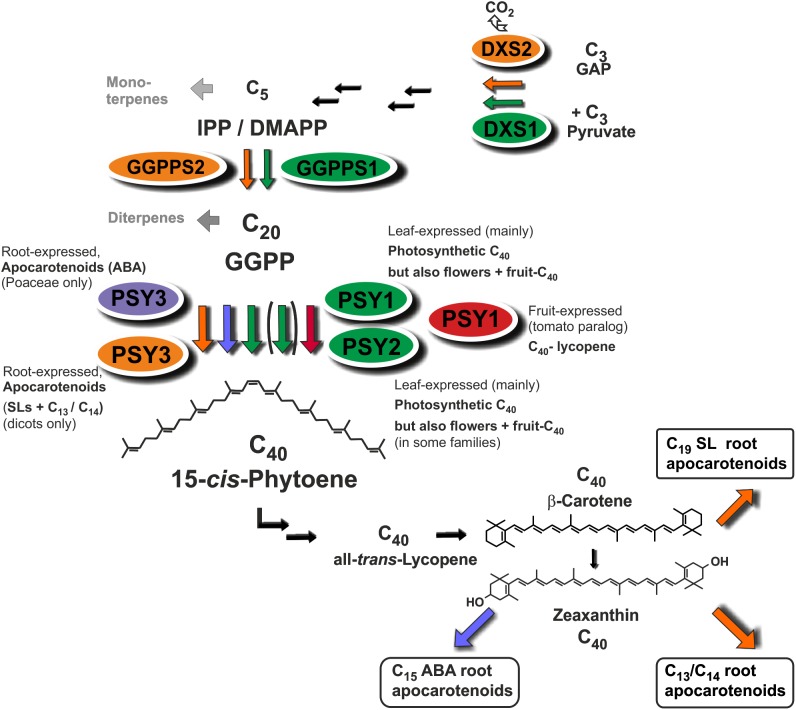
Roles of diversified PSY isoforms in carotenoid and apocarotenoid biosynthesis. PSY1 and PSY2 isoforms (marked in green and red) appear to be mainly involved in C_40_ carotenoid biosynthesis in leaves, flowers, and fruits. Two different types of PSY3 isoforms may be committed to integrative carotenoid/apocarotenoid biosynthesis in roots toward formation of boxed products such as ABA (PSY3 of Poaceae, marked in blue) or SL and C_13_/C_14_ apocarotenoids (PSY3 of dicots, marked in orange). Isoforms at upstream steps (DXS, GGPPS) are coregulated with PSY1/PSY2 (green background, DXS1, GGPPS1) or PSY3 of dicots (orange background, DXS2, GGPPS2) potentially forming separate product-oriented metabolons with the respective PSY isoforms.

*MtPSY3* and *SlPSY3* are not only coregulated with downstream steps to SL and C_13_/C_14_ apocarotenoids but also with at least two upstream steps at flux-controlling key positions in the MEP pathway for C_5_-IPP formation (*DXS*) and in C_5_ to C_20_ conversion (*GGPPS*). In both cases a coregulation profile with *MtPSY3* or *SlPSY3* matches with a particular member of diversified small gene families exhibiting differential expression (*DXS2*, *GGPPS2*; **Figure 7** and **Table 2**) as is the case for the *PSY* gene family. Together with the coregulated downstream steps these observations suggest a coregulated precursor supply chain pathway for symbiosis-supporting root apocarotenoids starting already with IPP formation in the MEP pathway (**Figure 8**). This supply chain pathway is dependent on uniquely AM-responsive isogenes or on those, which are AM-responsive in combination with other stimuli or housekeeping functions.

Since the above arguments for a regulated precursor supply chain pathway are based only on coregulation analyses and transcriptional responses, we also performed an experiment to show metabolic elevation of carotenoid pathway activity upon mycorrhization under conditions used for the transcript analyses. Even more pronounced than in previous experiments (Fester et al., 2002b, 2005) we were able to show a strong increase in phytoene, the substrate of PDS, in norflurazon-treated mycorrhized plants under conditions very favorable for strong mycorrhization (**Figure 6**). These observations are compatible with a pathway model for SL and C_13_/C_14_ apocarotenoid biosynthesis, which receives its carotenoid substrates not from regular carotenoid pools in roots but rather from a co-regulated precursor supply pathway.

Physical association at plastidial membranes of sequential steps in carotenogenesis termed multi-enzyme complexes and metabolons has been proposed (Fraser et al., 2000). Such concepts of channeling intermediates toward certain endproducts is known from other pathways but the concept has remained largely hypothetical for carotenogenesis (reviewed in Ruiz-Sola and Rodriguez-Concepcion, 2012; Nisar et al., 2015). Our findings add a new chapter to the issue of the still largely hypothetical metabolon formation in carotenogenesis by extending it to apocarotenoid formation. The strong and coordinate upregulation by mycorrhization of (iso)genes from the first step of the MEP pathway (DXS) to the second cleavage step of SL formation (CCD8) suggests the existence of specific metabolons for apocarotenoid biosynthesis, which may contain both carotenogenic enzymes and those catalyzing isomerization and cleavage steps toward apocarotenoids (**Figure 8**). Such integrated metabolons would also avoid a competition for carotenoid pools from different apocarotenoid pathways. Having a “just in time” delivery system of carotenoid precursors for apocarotenoid biogenesis would keep the carotenoid concentrations low and intermediates would show up in elevated concentrations only upon interrupting metabolite flux. Indeed, interrupting delivery of intermediates by the norflurazon treatment revealed a level of phytoene elevated up to 240-fold in mycorrhizal roots relative to negative controls (**Figure 6**).

### Evolution of the Plant PSY Gene and Protein Family: Photosynthetic and Non-photosynthetic Regulation and Functions

Research on the crtB/PSY gene and protein family and its diversification has a rich history, which we have embedded here into the timeline of evolution from photosynthetic bacteria, through algae to land plants (**Figure 4** and Supplementary Figure S5). Integrating a large number of sequences into a phylogenetic analysis of PSYs has revealed previously unrecognized clades of PSYs with highly differential expression profiles (**Figure 4**). Throughout evolution PSYs and their crtB ancestors have always played key roles in producing carotenoids as light-harvesting molecules and photoprotectants in photosynthetic cells and tissues. A major diversification of *PSY* genes is observed in angiosperms, apparently starting already in basal angiosperms before the separation into eudicots and monocots. The *A. trichopoda* genome contains two *bona fide PSY* genes, which may be the ancestors of the PSY1 and PSY2 “green” clades of monocots and dicots probably initially specialized for optimized photosynthetic functions. Such a separation into two “green” clades in contemporary angiosperms is obvious from the separation of three “green” *MtPSY* genes strongly expressed in leaves (*MtPSY1*, *MtPSY2a*, *MtPSY2b*; **Figure 4**). Within these two green clades there are single genes or paralogous “spin-offs,” which have more recently adopted expression in non-photosynthetic tissues, while largely loosing (*SlPSY1*; Giorio et al., 2008) or maintaining (*Daucus carota DcPSY1*, *DcPSY2*; Bowman et al., 2014; Wang et al., 2014) expression in photosynthetic tissues. PSYs conforming to clade 2 have thus far not been identified in solanaceous species and also not in Brassicaceae.

With the exception of carrots and other carotenoid-rich roots, members of the PSY1/PSY2 clades are expressed in roots only at low level or not at all. However, roots appear to have evolved two classes of specialized PSYs, which are committed to or at least preferentially expressed in the context of two specific apocarotenoid formation processes in roots, while still retaining some residual expression in other organs or by other cues. The first-discovered case is the PSY3 class of the Poaceae involved in increased ABA formation in response to abiotic stresses such as drought or salinity (Li et al., 2008; Welsch et al., 2008) also responding to ABA application in wheat roots (Dibari et al., 2012). Members of this class appear to have evolved rather recently from within the monocot PSY1 class (**Figure 4**).

A second case, studied here, is dPSY3, clearly separated in primary structure and also expression profiles from the Poaceae-PSY3 clade (**Figure 4** and Supplementary Figure S5). Based on current knowledge collected in this paper the main commitment of dPSY3s appears to be the biosynthesis of apocarotenoids supporting root symbioses such as the SLs but also the AM-induced C_13_/C_14_ apocarotenoids. By contrast, *MtPSY3* was not responsive to drought treatments under conditions, which resulted in higher transcript levels of an ABA biosynthesis marker gene (*MtNCED*; **Figure 2B**) suggesting that *MtPSY3* is likely not involved in ABA formation. In other studies ABA levels strongly decreased in drought-stressed tomato roots upon mycorrhization (Chitarra et al., 2016) probably because the AM fungal hyphae can deliver water to the roots and thus act in alleviating drought stress. This additional beneficial fungal activity reduces the need for ABA-mediated plant responses. In accordance with this view, the *MtNCED* marker was not responsive at all to mycorrhization (**Figure 2B**).

d*PSY3* genes appear to be of ancient origin, since they are most closely related to PSYs from early land plants and algae. Perhaps a common ancestor of higher plant PSYs diversified into (i) the current PSY1/PSY2 clades preferentially optimized for photosynthetic functions with spin-offs for fruit- and flower-specific expression and (ii) the dPSY3 clade. Such a scenario is supported by the finding of three PSYs in the basal angiosperm *A. trichopoda*, each of which can be tentatively assigned to one of the three clades (**Figure 4**). It is conceivable that dPSY3s co-evolved with the AM symbiosis, which first emerged some 400 Mio years ago and which still appears to be their major commitment as judged from their unique symbiont-activated expression profiles (**Figures 2**, **3** and Supplementary Figures S2, S3).

Different plant lineages have thus chosen different strategies of molecular evolution to optimize the bottleneck of phytoene synthesis in both aboveground carotenoid and belowground apocarotenoid biosynthesis. Evolution and selection seems to be driven into maintaining and optimizing old genes (e.g., d*PSY3*) but also into the constant generation of new duplicates for specialized roles (e.g., *SlPSY1*). There have been many attempts to get around the PSY bottleneck by biotechnology, i.e., by overexpression of *PSY* genes of various origins in transgenic plants. Those targeting non-green tissues have achieved superior success (Maass et al., 2009). Following along those lines, it might be worth overexpressing d*PSY3* genes or other *PSY* isogenes in a root-specific manner and/or driven by a strong AM-inducible promoter. This might overcome a likely bottleneck in SL formation and thus achieve an improvement of root colonization and mycorrhizal performance beyond wild-type plants to arrive eventually at agricultural practices relying on low-input plant mineral nutrition through a better support of root symbioses.

## Author Contributions

MW and AT designed the research. RS, RW, and MC performed the experiments. GB and WK contributed to analytical procedures. MW analyzed the data and wrote the article.

## Conflict of Interest Statement

The authors declare that the research was conducted in the absence of any commercial or financial relationships that could be construed as a potential conflict of interest.

## Footnotes

1 http://www.geneious.com/

2 http://mtgea.noble.org/v3/

3 http://mtgea.noble.org/v3/blast_search_form.php

4 https://solgenomics.net/

5 https://phytozome.jgi.doe.gov/pz/portal.html
