# Supplementary material for: Strigolactone Levels in Dicot Roots Are Determined by an Ancestral Symbiosis-Regulated Clade of the PHYTOENE SYNTHASE Gene Family
Source: Front Plant Sci. 2018 Mar 1;9:255. doi: 10.3389/fpls.2018.00255 (PMC5838088; doi:10.3389/fpls.2018.00255)
Supplement: Supplementary file 1 [file Table_1.pdf]

Supplementary Table 1

Accession Numbers or Gene IDs of Amino Acid Sequences Used in Figure 4 and in Supplementary Figures S4 and S5

Species	Designation and PSY type	Accession or ID
Acquilegia coerulea	AcPSY3	Aquca_009_00453
Amborella trichopoda	AtrPSY1	evm_27.TU.AmTr_v1.0_scaffold00032.92
Amborella trichopoda	AtrPSY2	evm_27.TU.AmTr_v1.0_scaffold00044.14
Amborella trichopoda	AtrPSY3 (truncated, 190 aa)	evm_27.TU.AmTr_v1.0_scaffold00133.50
Arabidopsis thaliana	AtPSY	At5g17230
Brachypodium distachon	BdPSY1	Bradi1g29590
Brachypodium distachon	BdPSY2	Bradi4g01100
Brachypodium distachon	BdPSY3	Bradi4g37520
Brassica napus	BnPSY1a	KF297333
Brassica napus	BnPSY1b	KF297332
Brassica napus	BnPSY1c	KF297331
Capsicum annuum	CaPSY1	P37272 (PSY_CAPAN)
Chlamydomonas reinhardtii	CrPSY	XP_001701192
Citrus sinensis	CsPSY1	orange1.1g044623m
Citrus sinensis	CsPSY3a (formerly CsPSY2)	orange1.1g016696m
Citrus sinensis	CsPSY3b (formerly CsPSY3)	orange1.1g036368m
Cucumis melo	CmPSY1	JF745118
Cucumis melo	CmPSY3 (formerly CmPSY2)	JF745117
Daucus carota	DcPSY1	DQ192186
Daucus carota	DcPSY2	DQ192187
Daucus carota	DcPSY3	XP_017217851
Dunaliella bardawis	DbPSY	U91900
Eriobotrya japonica	EjPSY1	KF922363.1
Eriobotrya japonica	EjPSY2a	KF922364.1
Eriobotrya japonica	EjPSY2b	KF922366.1
Eriobotrya japonica	EjPSY3	KF922367.1
Fragraria vesca	FvPSY1	gene31674-v1.0-hybrid
Fragraria vesca	FvPSY2	gene28765-v1.0-hybrid
Fragraria vesca	FvPSY3	gene24795-v1.0-hybrid
Glycine max	GmPSY1	Glyma.02G240200
Glycine max	GmPSY2a	Glyma.14G031200
Glycine max	GmPSY2b	Glyma.18G111900
Glycine max	GmPSY3	Glyma.18G000600
Haematococcus fluvialis	HfPSY	DQ057355
Lotus japonicus	LjPSY1	Lj2g3v2507910.2
Lotus japonicus	LjPSY2	Lj0g3v0184729.2
Lotus japonicus	LPSY3	Lj0g3v0278879.2
Malus domestica	MdPSY2a	MDP0000177623
Malus domestica	MdPSY2b	MDP0000237124
Malus domestica	MdPSY3	MDP0000151924
Manihot esculenta	MePSY1	Manes.02G081700.1
Manihot esculenta	MePSY2 (PSY1-type)	Manes.01G124200.1
Manihot esculenta	MePSY3	Manes.03G084700.1

Medicago truncatula	MtPSY1	Medtr5g076620
Medicago truncatula	MtPSY2a	Medtr3g450510
Medicago truncatula	MtPSY2b	Medtr5g090780
Medicago truncatula	MtPSY3	Medtr3g083630
Musa acuminata	MaPSY1a	GSMUA_AchrUn_randomG09240_001
Musa acuminata	MaPSY1b	GSMUA_Achr11T07450_001
Musa acuminata	MaPSY2a	GSMUA_Achr9G10050_001
Musa acuminata	MaPSY2b	GSMUA_Achr6G31560_001
Oryza sativa	OsPSY1	LOC_Os06g51290
Oryza sativa	OsPSY2	LOC_Os12g43130
Oryza sativa	OsPSY3	LOC_Os09g38320
Panicum hallerii	PhPSY3	Pahal.B03778
Pantoea ananatis	PacrtB	D90087
Phaseolus vulgaris	PvPSY1	Phvul.008G241500
Phaseolus vulgaris	PvPSY2a	Phvul.006G024100
Phaseolus vulgaris	PvPSY2b	Phvul.008G195800
Phaseolus vulgaris	PvPSY3	Phvul.001G268600
Physcomitrella patens	PpPSY1a	Pp3c24_10180
Physcomitrella patens	PpPSY1b	Pp3c24_16380
Physcomitrella patens	PpPSY1c	Pp3c8_21760
Picea abies	PaPSY	EF676374.1
Ricinus communis	RcPSY3	29835.t000015
Selaginella moellendorfii	SmPSY	233658<br>(alias fgenesh1_pm.C_scaffold_47000028)
Setaria italica	SiPSY3	Seita.2G303000
Solanum lycopersicum	SlPSY1	Solyc03g031860
Solanum lycopersicum	SlPSY2 (PSY1-type)	Solyc02g081330
Solanum lycopersicum	SlPSY3	Solyc01g005940
Sorghum bicolor	SbPSY1	Sobic.010G276400
Sorghum bicolor	SbPSY2	Sobic.008G180800
Sorghum bicolor	SbPSY3	Sobic.002G292600
Spirodela polyrhiza	SpPSY1	Spipo1G0051600
Spirodela polyrhiza	SpPSY2	Spipo8G0020300
Vitis vinifera	VvPSY3	XP_010651361
Zea mays	ZmPSY1	GRMZM2G300348
Zea mays	ZmPSY2	GRMZM2G149317
Zea mays	ZmPSY3	ACG30201
