# Supplementary material for: Strigolactone Levels in Dicot Roots Are Determined by an Ancestral Symbiosis-Regulated Clade of the PHYTOENE SYNTHASE Gene Family
Source: Front Plant Sci. 2018 Mar 1;9:255. doi: 10.3389/fpls.2018.00255 (PMC5838088; doi:10.3389/fpls.2018.00255)
Supplement: Supplementary file 2 [file Table_2.pdf]

Supplementary Table 2

List of Primers Used for qRT-PCR and Cloning Presented in 5'- 3' Direction

qRT-PCR

M. truncatula

MtCCD7	fwd	ATG CAA GCC AAG CCC ATT CCA
	rev	TTC GGT ATG GGC GTG GAT ATT G
MtCCD8	fwd	GAC TAC AAC TTC AGG CAC CTC T
	rev	ATA AGC TTG AGA TTC AAC TTG CCG
MtDXS1	fwd	CCG ATT CCC AAC AAG ATG CTT
	rev	GGT CAT AGG CCC TTT GCA AGA
MtDXS2	fwd	CAC CTT GGA TAC ATA AAT CAT TAA GTC TCT
	rev	CCG AAT CTC TTC TCT CAA CCA AGA
MtEF	fwd	AGA AGG AAG CTG CTG AGA TGA AC
	rev	TGA CTG TGC AGT AGT ACT TGG TG
MtNCED	fwd	TGA CTT AGC AGC GAC TCG TTC
	rev	CTC GAA CAA ACC AAG AAC GAT TC
MtPSY1	fwd	TTG AGG AAT AGG AGG AAG TTA AGG
	rev	AAG CTT CAC AAC CCC GAA AGA ATC
MtPSY2a	fwd	TTG TTA GGG TGA ATT GTG ATG CAA A
	rev	CCC CAC TTC CAA ACC TTA CC
MtPSY2b	fwd	TTC TTT GGG TGA ATT GTG GAC TCA
	rev	CAA GCA AAG CTA ATT CCA GAA CAA TG
MtPSY3	fwd	GGC TAG CGT TAT TTG TTT ATT GTG C
	rev	TCA TTT CTG GAA CTG AAG CCA AGT

S. lycopersicum

SlCCD7	fwd	CTG AAT GGA ACA AAG CAG CA
	rev	CCG CGT TTA ATT TCA CGA CT
SlCCD8	fwd	CAG TGG CAA CAT TGT GGC AAG T
	rev	GTG GTG TCT GCG CTA TGC TC
SlD27	fwd	AGG CTG CAA GGG TTG TGT AT
	rev	CAA GGT CCA ACT AGC CAA GG
SlDXS1	fwd	AAG ATG GAA GAA GCG TAG AAA GA
	rev	TCA AGA ACC GGT TAC ACA ATC A
SlDXS2	fwd	TCT ACG GAT CGA TGC CTT CT
	rev	TGC AAT ATG ATC ATC ACT ACA TGA
SlEF	fwd	GCT GCT GTA ACA AGA TGG ATG C
	rev	AGG GTT GTA ACC AAC CTT CTT GAG
SlGGPPS1	fwd	CTC CCA CCA AGC CAA TCT TA
	rev	CCT TGA TTG CCT TCG TTA CC
SlGGPPS2	fwd	CGT AGC TGA AAA GGC GAT TTG
	rev	CGC CAT GGC ATT TCC TTG G
SlPSY1	fwd	GAG AGA ATC AAT AGA GGT GGT GG
	rev	CCG GCT TCA CTT CTA ACT CAT TG
SlPSY2	fwd	GAA GAT CAA GAA CTG AGA ATG GAAG

	rev	CCG GCT TCA CTT GTA TGT CAT C
SlPSY3	fwd	GCC TAG TTT AGC CAT TCA ATA GAC
	rev	ATG GTA TGC TTT GAA GTT CAC AAG C

Rhizophagus irregularis

RiBTUB	fwd	TGG TGC AGG AAA CAA TTG GGC CA
	rev	AGC TCC AGT ACC ACC GCC GA

Cloning RNAi fragment

*MtPSY3*_sense	fwd	TTG GTC TCA AAT GCC GGT TTG GTC CTC ATT AAT ATT GTA TCG CA
	rev	TTG GTC TCA ACC TGC ACA ATA AAC AAA TAA CGC TAG CCT TTG GG
MtPSY3_Asense	fwd	TTG GTC TCA CAG GGC ACA ATA AAC AAA TAA CGC TAG CCT TTG GG
	rev	TTG GTC TCA AAG CCC GGT TTG GTC CTC ATT AAT ATT GTA TCG CA
