# Supplementary figures and images for: Strigolactone Levels in Dicot Roots Are Determined by an Ancestral Symbiosis-Regulated Clade of the PHYTOENE SYNTHASE Gene Family
Source: Front Plant Sci. 2018 Mar 1;9:255. doi: 10.3389/fpls.2018.00255 (PMC5838088; doi:10.3389/fpls.2018.00255)

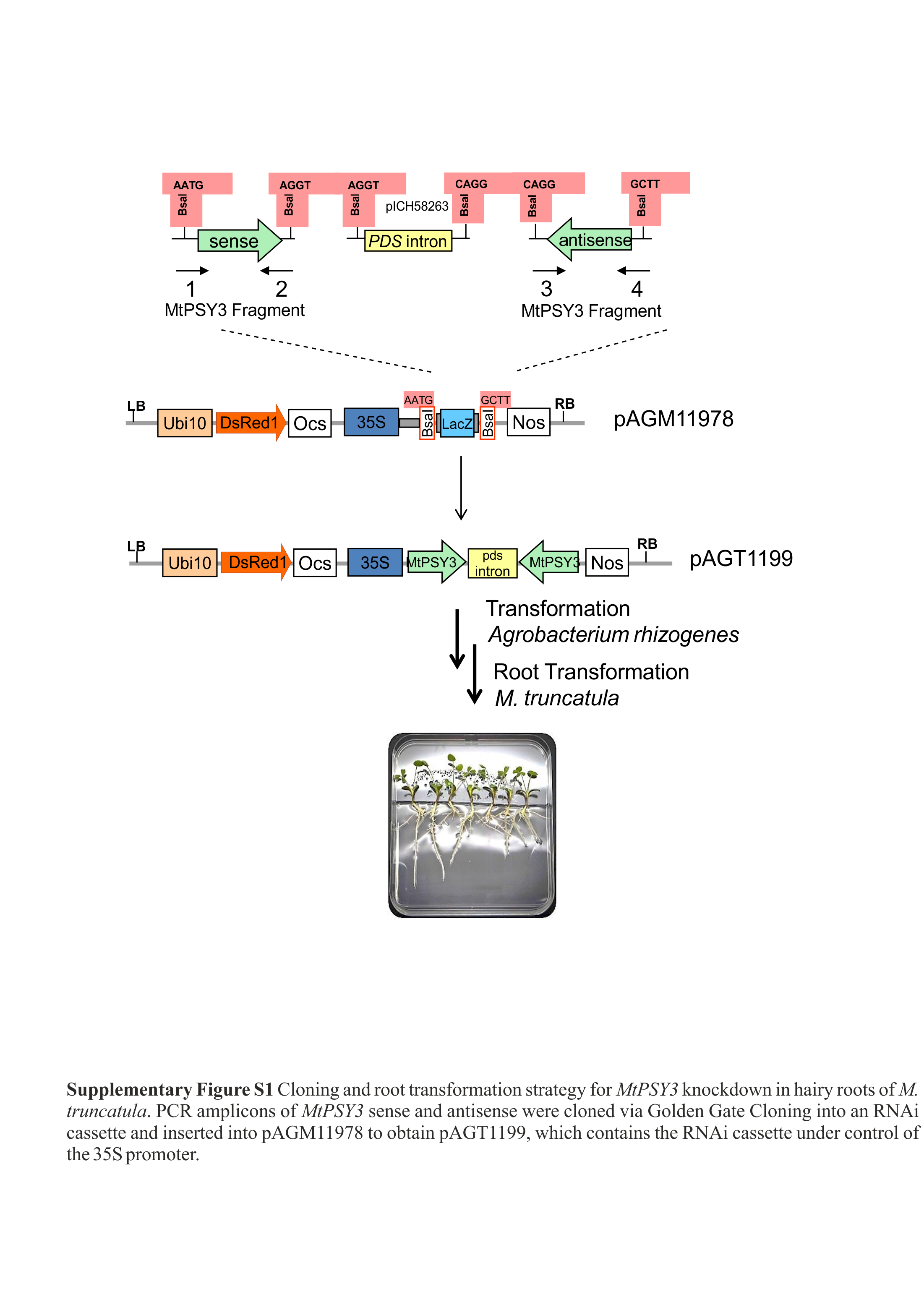

Supplement: Supplementary file 3 [file Image_1.tif]

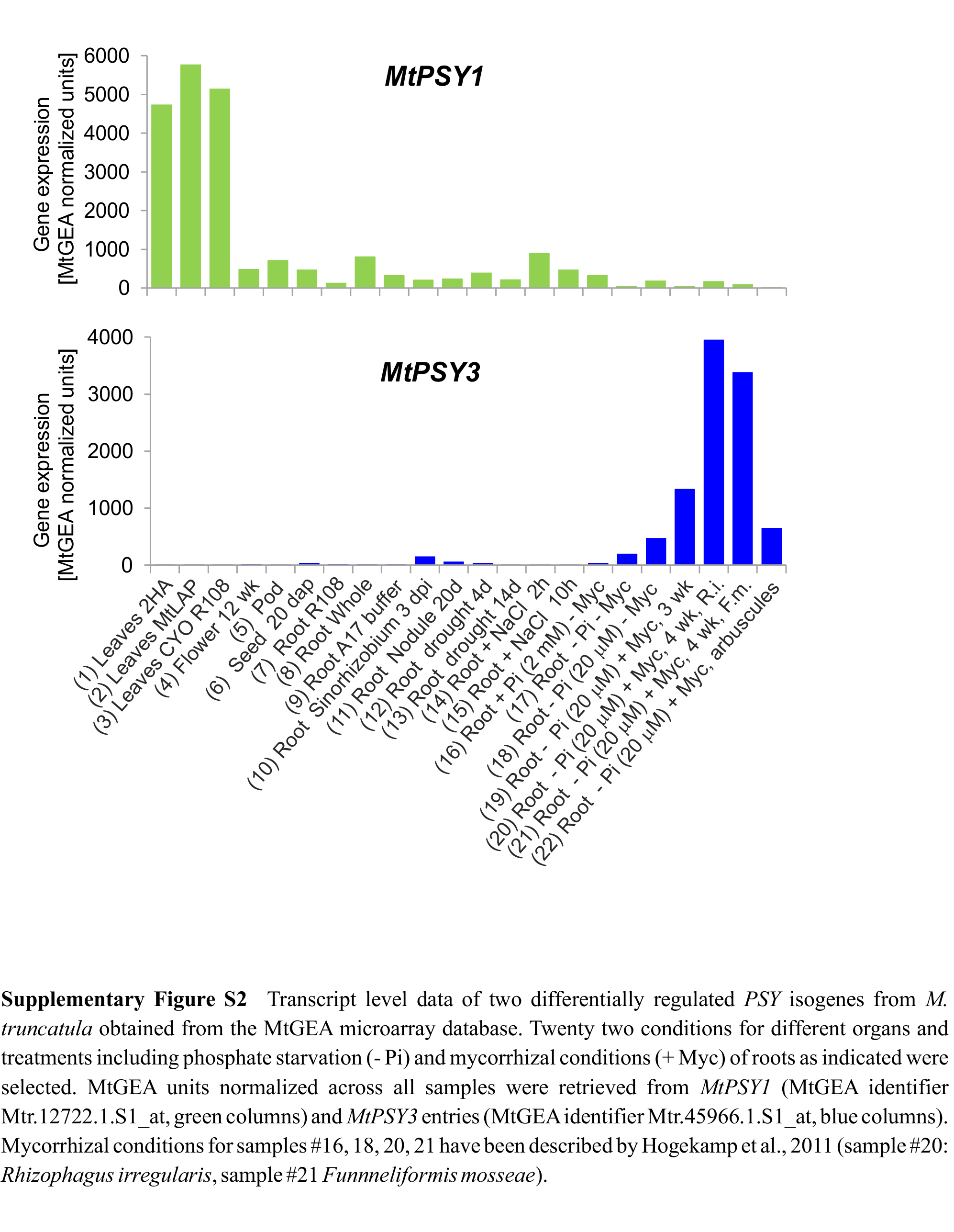

Supplement: Supplementary file 4 [file Image_2.tif]

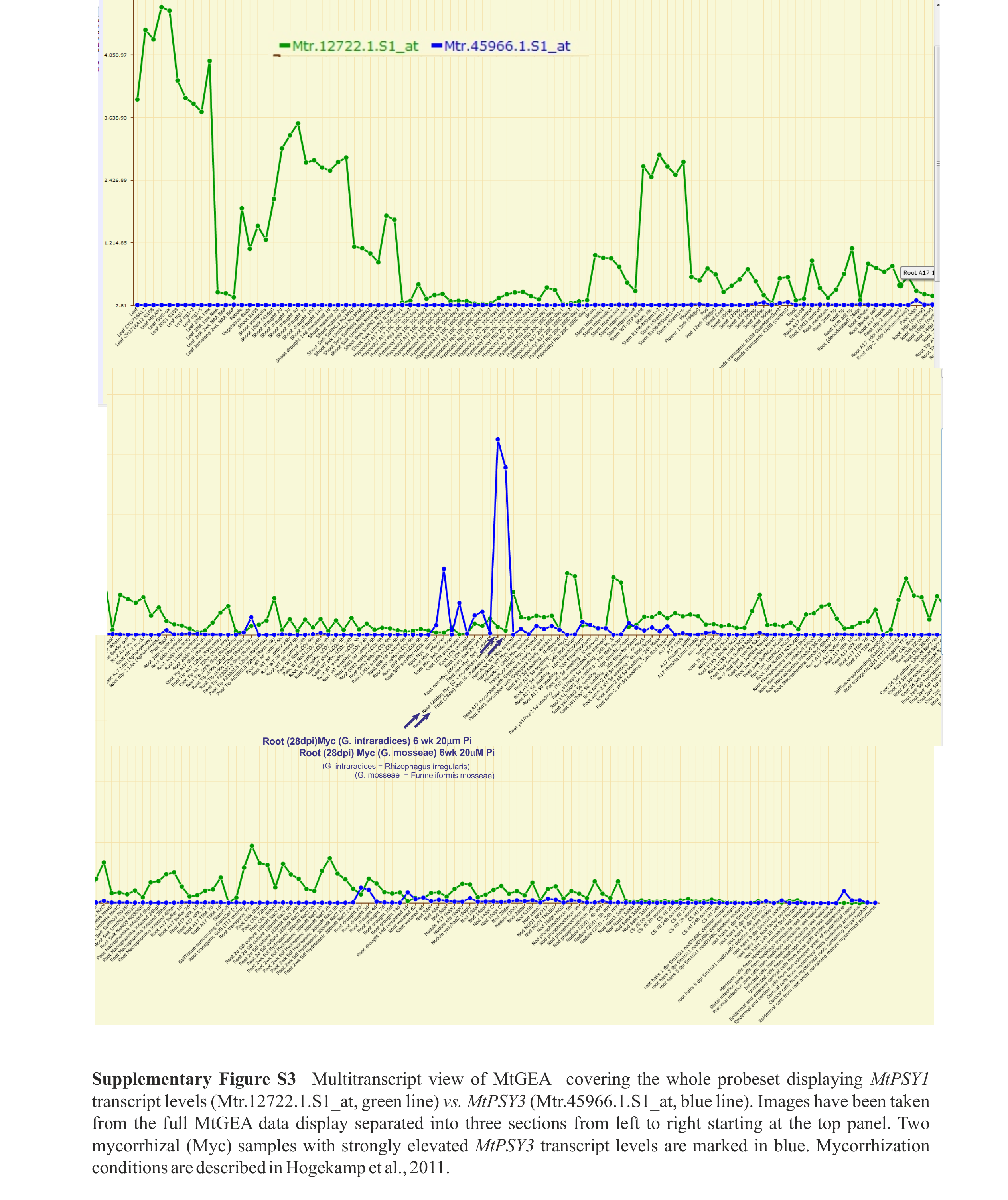

Supplement: Supplementary file 5 [file Image_3.tif]

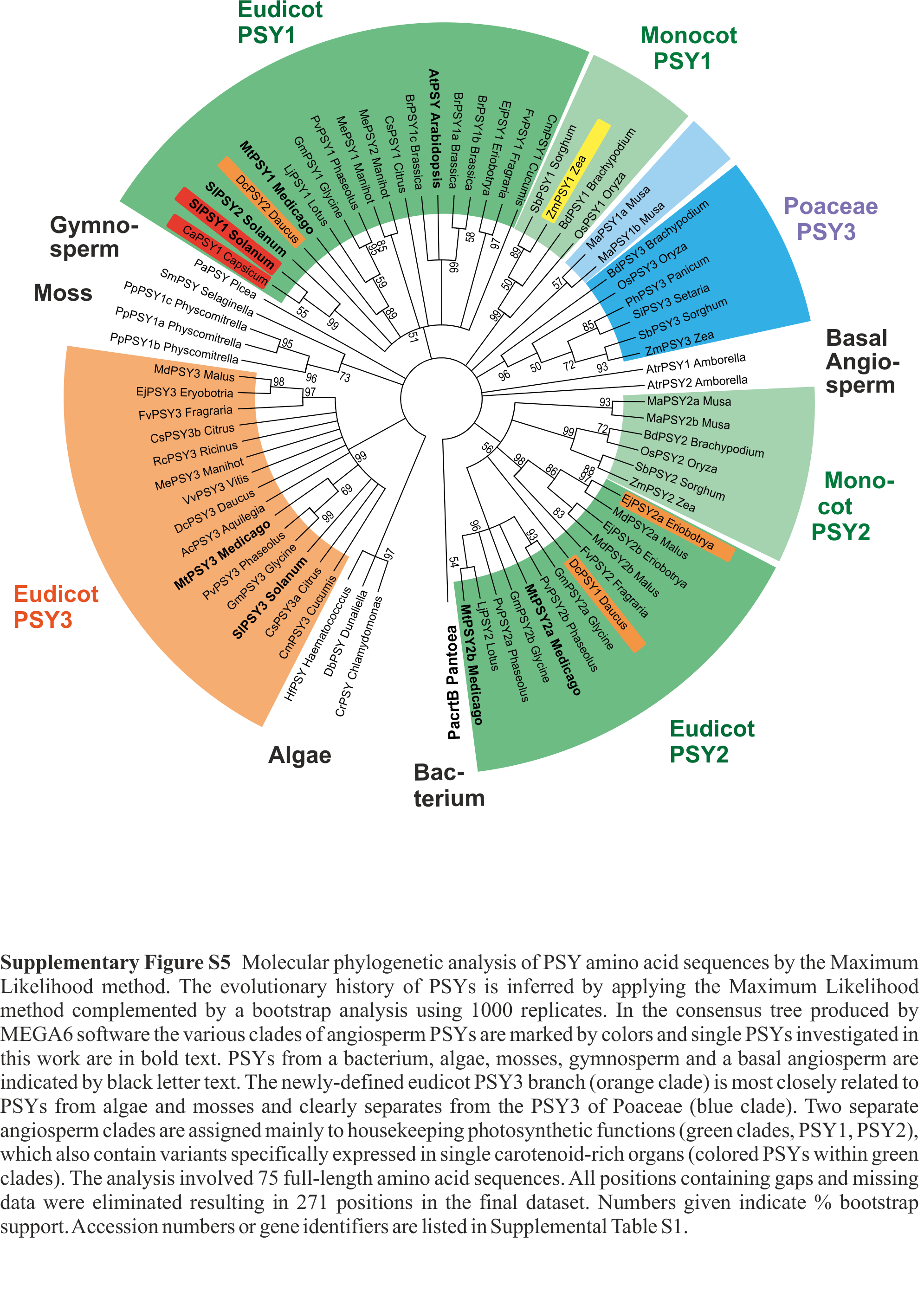

Supplement: Supplementary file 7 [file Image_5.tif]

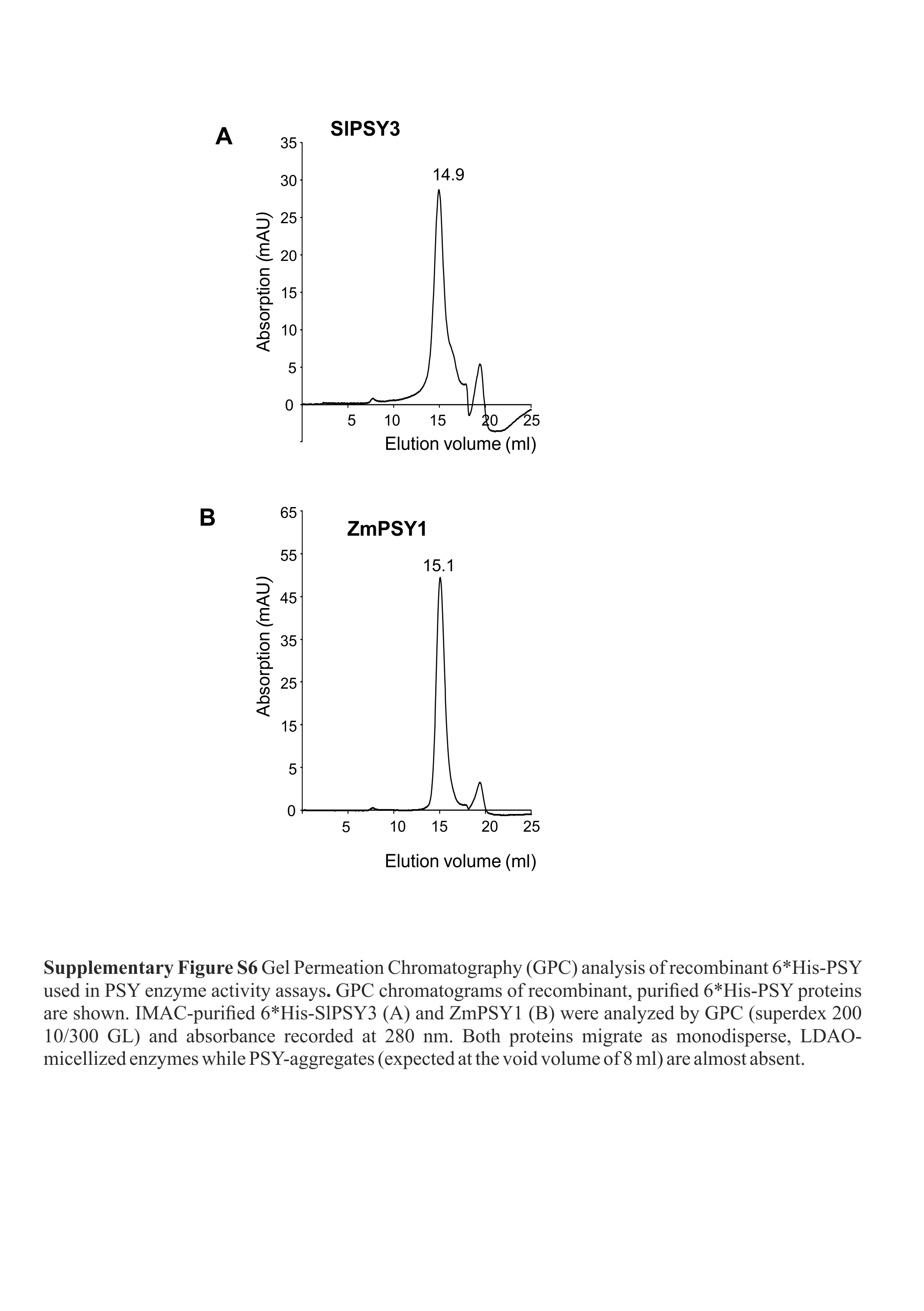

Supplement: Supplementary file 8 [file Image_6.tif]

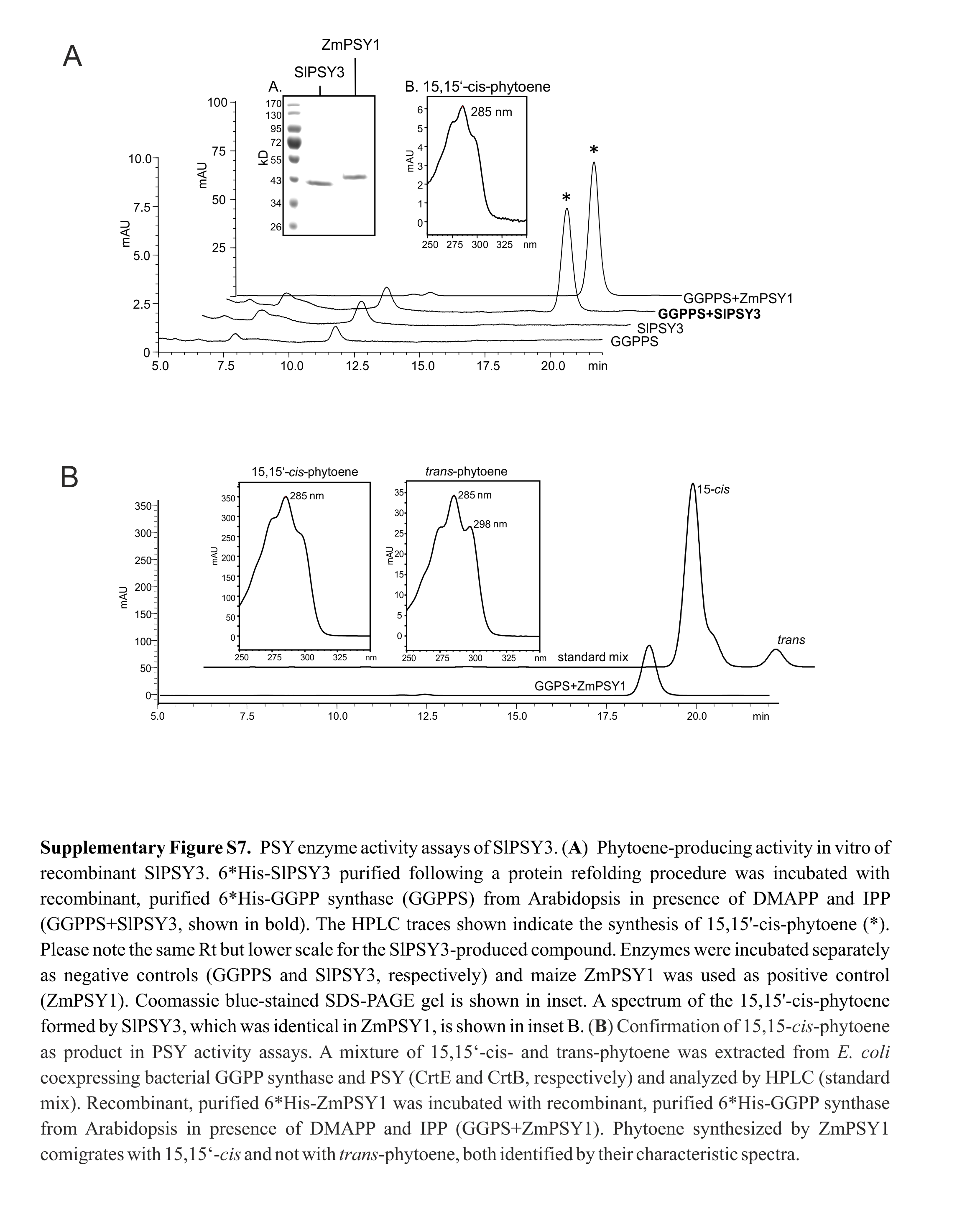

Supplement: Supplementary file 9 [file Image_7.tif]

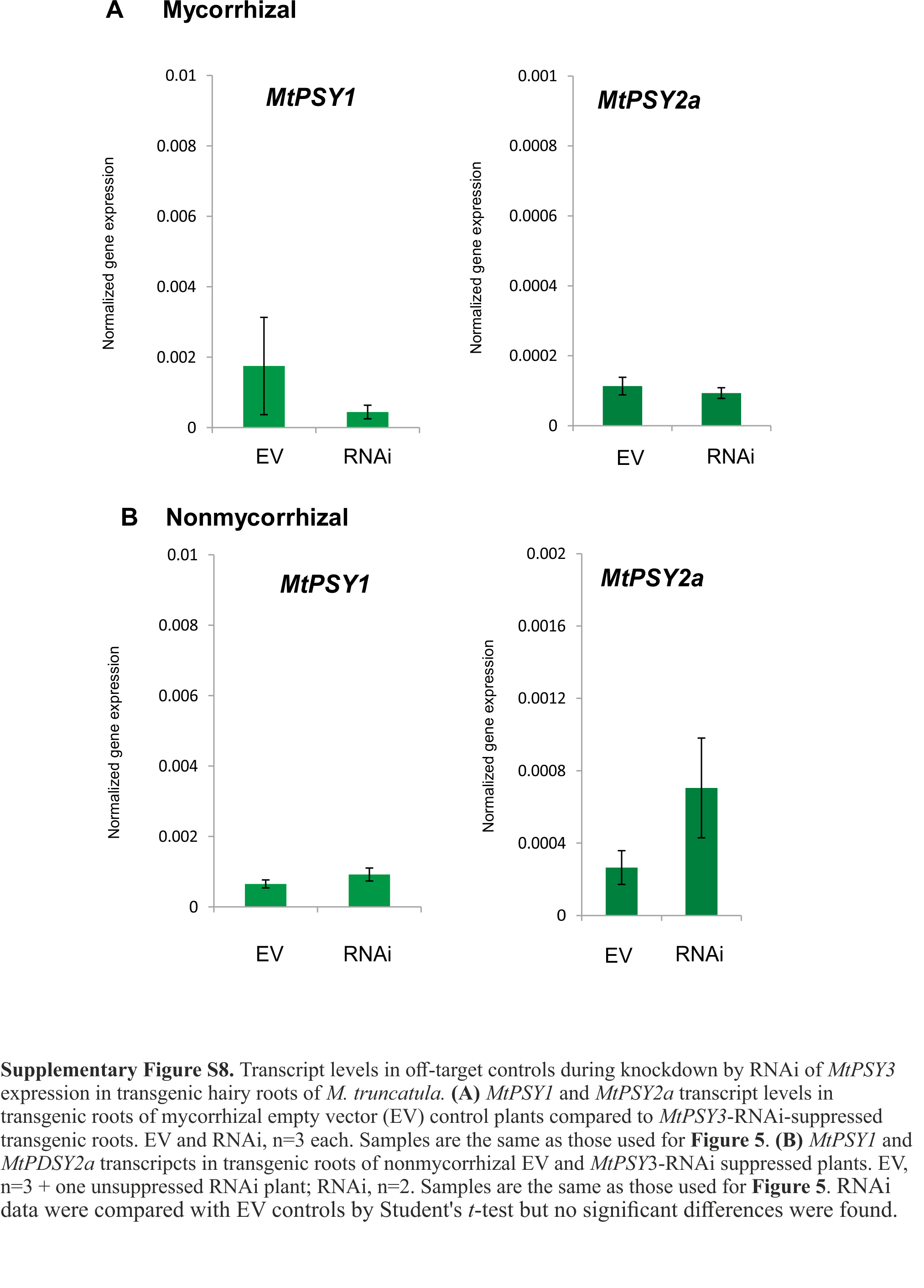

Supplement: Supplementary file 10 [file Image_8.tif]
