# Supplementary material for: Strigolactone Levels in Dicot Roots Are Determined by an Ancestral Symbiosis-Regulated Clade of the PHYTOENE SYNTHASE Gene Family
Source: Front Plant Sci. 2018 Mar 1;9:255. doi: 10.3389/fpls.2018.00255 (PMC5838088; doi:10.3389/fpls.2018.00255)
Supplement: Supplementary file 6 [file Image_4.pdf]

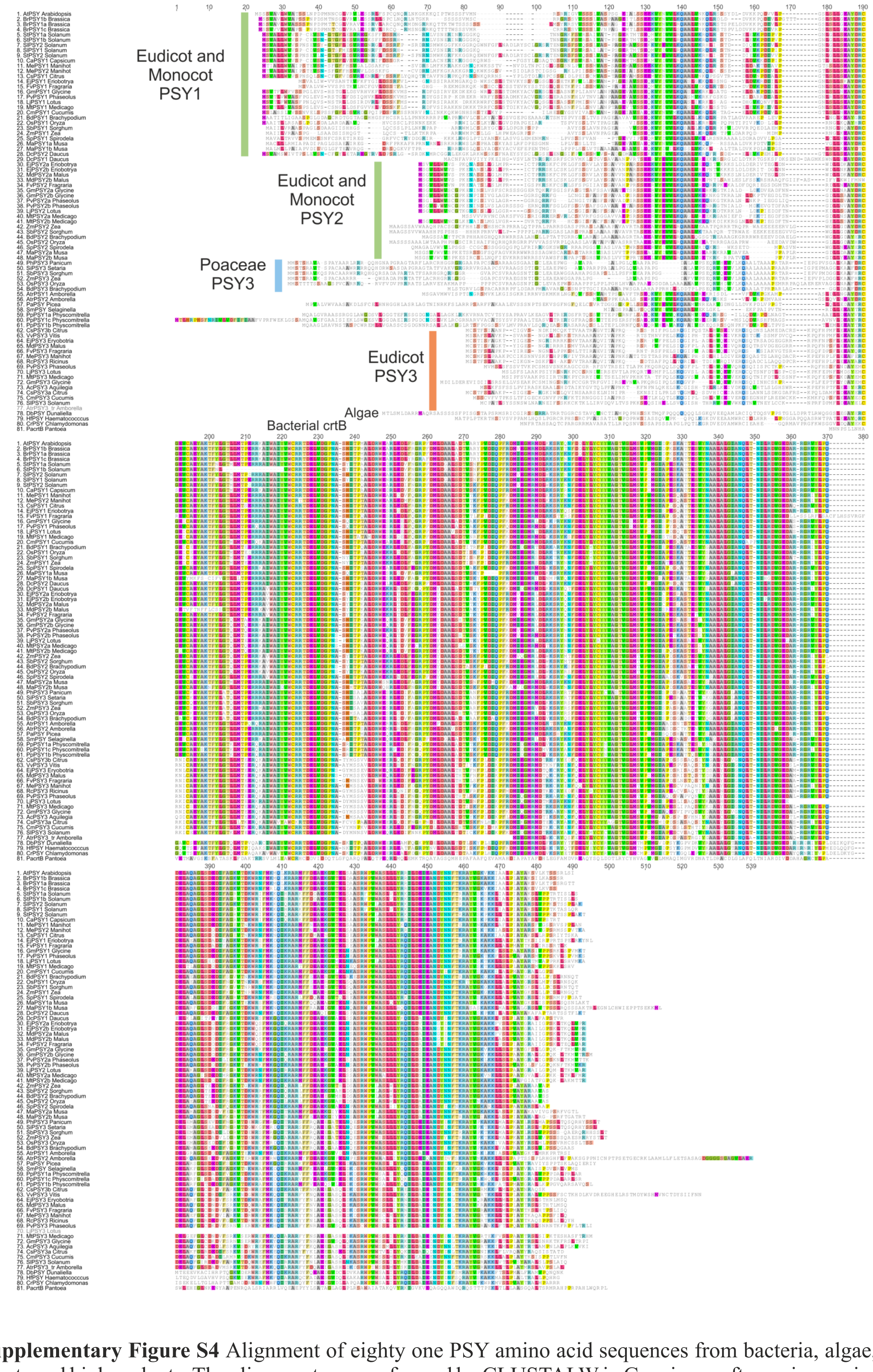

Supplementary Figure S4 Alignment of eighty one PSY amino acid sequences from bacteria, algae, primitive plants and higher plants. The alignment was performed by CLUSTALW in Geneious software increasing gap open penalty to 20 and gap extension penalty to 10 in order to reduce extensive gaps. Colors highlight agreements with consensus. Sequences were ordered manually to move sequences from more ancient organisms to the bottom and group related sequences. The various clades of angiosperm PSYs are marked by colored vertical bars to indicate the different sizes of their N-termini. Accession numbers and gene identifiers are given in Table S1.
